# Barriers and Facilitators to the Development and Implementation of Public Policies Addressing Food Systems in Five Sub-Saharan African Countries and Five of Their Cities

**DOI:** 10.34172/ijhpm.8592

**Published:** 2025-03-18

**Authors:** Celia Burgaz, Iris Van Dam, Adama Diouf, Kouakou Philipps Kouakou, Olouwafemi M. Mama, Sabiba Kou’santa Amouzou, Rebecca Rachel Assa Yao, Blessing Atwine, Madina M. Guloba, Lallepak Lamboni, Pauline Nakitende, Julien S. Manga, Clémence Metonnou, Célestin Koffi N’dri, Reynald Santos, Charles Sossa, Papa M.D.D. Sylla, Tiatou Souho, Stefanie Vandevijvere

**Affiliations:** ^1^Department of Epidemiology and Public Health, Sciensano, Brussels, Belgium.; ^2^Department of Geosciences, Environment and Society, Université libre de Bruxelles (ULB), Brussels, Belgium.; ^3^Laboratoire de Recherche en Nutrition et Alimentation Humaine (LARNAH), Université Cheikh Anta Diop, Dakar, Senegal.; ^4^Université Alassane Ouattara (UAO), Bouaké, Côte d’Ivoire.; ^5^Laboratoire de Biochimie des Aliments et Nutrition, University of Kara, Kara, Togo.; ^6^Economic Policy Research Centre (EPRC), Kampala, Uganda.; ^7^Department of Nutrition, University of Montreal, Montreal, QC, Canada.; ^8^Regional Institute of Public Health, Université of Abomey-Calavi (UAC), Ouidah, Benin.; ^9^Laboratoire des Sciences Biologiques, Agronomiques, Alimentaires et de Modélisation des Systèmes Complexes (LABAAM), Université Gaston Berger de Saint-Louis, Saint-Louis, Senegal.

**Keywords:** Food Systems, Global Syndemic, Policy, Sub-Saharan Africa

## Abstract

**Background:**

There is increasing recognition of the role governments play in addressing the health and environmental sustainability challenges within current food systems. This study seeks to understand food system policies designed and/or implemented by selected national and local governments in Africa, and the barriers and facilitators faced when designing or implementing policies to create healthy and environmentally sustainable food systems.

**Methods:**

From an evidence-based list of proposed policies with double- or triple-duty potential to achieve healthy and environmentally sustainable food systems, a policy mapping was performed in five African countries (Benin, Côte d’Ivoire, Senegal, Togo, and Uganda) and one city in each of these countries (Ouidah, Bouaké, Saint-Louis, Sokodé, and Mbale). Semi-structured interviews were then conducted with policy stakeholders. The interview data were analysed in NVivo 14 using the thematic framework analysis approach, informed by the Health Policy Triangle (HPT).

**Results:**

The mapping showed that African countries have designed and implemented policies that simultaneously address food insecurity and climate change, mainly through food production policies. Within food environments, countries are focussing on interventions to prevent obesity, mainly food provision or food pricing policies. However, many policy gaps remain. Several technical and political barriers were commonly experienced when designing and implementing food system policies, regardless of the jurisdiction, context or region, such as insufficient financial resources, lack of political will, limited data, and inadequate monitoring and enforcement mechanisms. The major facilitators perceived were supportive public opinion and awareness, international agreements, sound agenda-setting, multi-sector and multi-stakeholder consultations and partnerships, availability of both financial resources and data, and solid political will.

**Conclusion:**

This article gives an overview of policies designed and implemented to achieve sustainable food systems, highlighting a strong focus through agriculture on undernutrition and climate change objectives. It also identifies their potential legislative, financial, and practical barriers and facilitators.

## Introduction

**Key Messages**

**Implications for policy makers**

- This article provides an overview of policies designed and implemented in selected African countries towards achieving healthier, more environmentally sustainable food systems.
- Some best practice policy examples are highlighted.
- It also identifies their potential legislative, financial, and practical barriers and facilitators perceived by different policy stakeholder groups towards the development and implementation of food system policies.

**Implications for the public**

This study provides an overview of the policy landscape of food systems in some selected African countries and cities, showing that governments are advancing towards policies that simultaneously address food insecurity and climate change, mainly through agriculture. Countries are also designing and implementing some policies to prevent obesity, mainly related to increasing the accessibility and availability of food. However, many policy gaps remain. Our results also highlight technical and political barriers that can be experienced when designing and implementing food system policies. For instance, insufficient financial resources, lack of political will, limited data, and inadequate monitoring and enforcement mechanisms. On the other hand, the major facilitators were: supportive public opinion and awareness, international agreements, sound agenda-setting, multi-sector and multi-stakeholder consultations and partnerships, availability of both financial resources and data, and solid political will.

It is well established that the current state of food systems is unsustainable from a human and planetary health perspective.^1-3^ Around the world, food systems are facing a triple challenge associated with the Global Syndemic of undernutrition, obesity and climate change.^4^ In 2020, 9.3% of the world’s population was affected by hunger,^5^ while 14% of the population suffered from obesity.^6^ In that same year, global greenhouse gas emissions amounted to more than 47 billion metric tons of carbon dioxide equivalent,^7^ contributing to climate change and environmental degradation. These three global pandemics affect most people in every country and region worldwide.^4^ The current functioning of food systems has been identified as the core factor behind these global, interconnected pandemics.^2^ Generally, policies tend to have a singular focus on specific elements and outcomes of the food system (eg, greenhouse gas emissions, food security, obesity rates) instead of having a broader, more holistic approach to improve simultaneously human and planetary health.^8,9^ Therefore, an urgent food system transformation is needed, with double and triple-duty actions towards healthy and sustainable food systems that guarantee food security and adequate healthy nutrition for all, in an environmentally sustainable way. There is increasing recognition of the important role that national and local governments can play in food system transformation,^10^ and in approaches and levers for change involving governments to deliver effective actions.^11^ A previously conducted scoping review showed that sustainable agriculture practices and school food programmes represent successful examples of policies that may simultaneously improve population nutrition and planetary health.^12^ In addition, some food labelling, reformulation, in-store nudging interventions and fiscal measures implemented worldwide have improved undernutrition and obesity.^12^ However, several research gaps exist in the effectiveness of policy interventions to tackle the Global Syndemic (ie, showing significant beneficial effects) across ten prior identified food system areas: (1) food production, (2) food processing, packaging and distribution, (3) food loss and waste, (4) food trade, (5) food composition, (6) food labelling, (7) food promotion, (8) food provision, (9) food retail, and (10) food prices.^12^

 While other regions of the Global South face urban transitions, Africa’s demographic shift raises important challenges to ensure that food systems guarantee optimal development and health outcomes for the population. Whilst the continent faces a growing problem of rising obesity trends,^13^ according to the latest data from 2023, Sub-Saharan Africa (SSA) faces a food crisis of unparalleled proportions caused by the effects of the war in Ukraine, the effects of climate change and the economic and social after-effects of the COVID-19 pandemic.^14^ Over the last years, the rates of food insecurity and undernutrition worsened, with nearly 282 million people in Africa undernourished in 2022, and an estimated 140 million people in the continent faced acute food insecurity.^15^

 However, African governments have committed to international pledges, such as the Malabo target of ending hunger and all forms of malnutrition by 2025, or the food security and nutrition targets of the Sustainable Development Goal 2 (SDG2) on Zero Hunger for 2030,^16^ that are supporting governments to work on solutions towards sustainable food systems. Yet, the most recent United Nations’ SDGs Report 2024 highlights that current progress falls far short of what is required to meet the SDGs by 2030.^17^ While recent studies show the progress made by governments in SSA towards improving populations’ health,^18,19^ little evidence is known about all the policy actions undertaken towards healthy and environmentally sustainable food systems.

 To address this gap, the aims of this study are: (1) to explore which policies toward healthy and environmentally sustainable food systems have been designed and/or implemented in selected national and local governments in SSA (Benin, Côte d’Ivoire, Senegal, Togo, and Uganda), and (2) to identify the barriers and facilitators faced by relevant policy stakeholders during the design or implementation of policies to create healthy and environmentally sustainable food systems.

## Methods

 Written informed consent was gathered from all stakeholders participating in the semi-structured interviews.

###  Identification of Policies 

 Policies with double- or triple-duty action were previously identified through a compilation of international policy recommendations, a scoping review,^12^ and international expert consultations.^20^ Policies were considered to have double- or triple-duty action if they were deemed effective (ie, showed significant beneficial effects) in tackling two or three of the following outcomes: (*a*) undernutrition, (*b*) obesity/non-communicable diseases (NCDs), and (*c*) climate change. Their full definition is available in Supplementary file 1.

**Figure 1:**
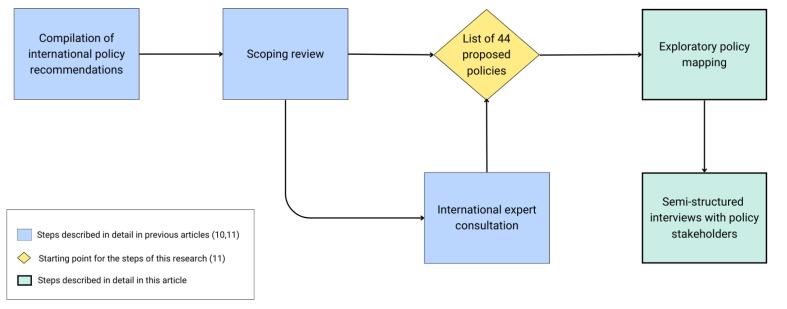
Key Elements of the Full Research Project and the Parts Related to This Study. Source: Author’s depiction.

 Based on these steps, a list of 44 proposed policies to create healthy and environmentally sustainable food systems was generated, establishing the basis for a Food System Policy Index including policies with double- and triple-duty potential to evaluate government actions.^20^ The key elements of the process followed to create the proposed policies are depicted in Figure 1.

 The Policy Index was divided into two main policy domains: “food supply chains” and “food environments,”^20^ which in turn were divided into a total of 10 subdomains: (1) food production, (2) food storage, processing, packaging and distribution, (3) food loss and waste, (4) food trade, (5) food composition, (6) food labelling, (7) food promotion, (8) food provision, (9) food retail, and (10) food prices. Their definition is available in Supplementary file 1.

###  Selection of Countries and Cities

**Figure 2:**
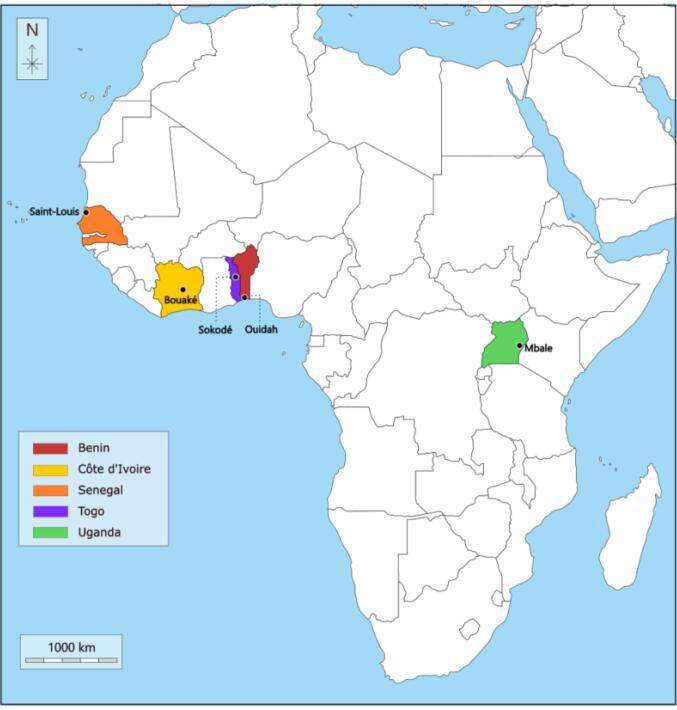
African Countries and Cities Included in This Study. Source: Author’s depiction adapted from the base map available at d-maps.com.

 The countries involved in this study were part of the INFORMAS 2.0 project,^21^ which included project partners from East and West Africa with available resources to research food systems. Each country team chose one city based on either the prevalence of malnutrition in the city/region or relevant agroeconomic activities (eg, agriculture, fisheries, strategic geographic location for trade) of the specific city/region when compared to other cities/regions within the respective countries. The exploratory policy mapping and the interviews with policy stakeholders covered five countries (Benin, Côte d’Ivoire, Senegal, Togo, and Uganda) and a city within each country (Ouidah, Bouaké, Saint-Louis, Sokodé, and Mbale) (Figure 2).

 In the period of the data collection (2022-2023), the political direction and the political parties in power of the countries analysed were as follows:

- Benin: Left-wing (*Progressive Union*)
- Côte d’Ivoire: Right-wing/liberal (*Rally of the Republicans*)
- Senegal: Left-wing (*African Patriots of Senegal for Work, Ethics and Fraternity*)
- Togo: Right-wing (*Union for the Republic*)
- Uganda: Right-wing (*National Resistance Movement*)

Between 2020 and 2022, the prevalence of the undernourished population was 10% in Benin, 7.7% in Côte d’Ivoire, 5.7% in Senegal, 17.4% in Togo and 31.6% in Uganda.^22^ Based on data from the World Bank,^23^ the domestic sectors of agriculture, forestry and fishing contributed more than 25% to Benin’s gross domestic product (GDP) of Benin, more than 14% to Côte d’Ivoire’s GDP, more than 16% to Senegal’s GDP of Senegal, 18% to Togo’s GDP and almost 24% to Uganda’s GDP in 2023.

###  Data Collection and Analysis

####  Policy Mapping

 The policy mapping builds on a systematic approach using the policy domains and subdomains proposed by the Food Systems Policy Index.^20^ Its scope was to identify food system policies designed and/or implemented by governments across the countries and cities selected. A broad view of policy was taken, including all government policies, interventions, plans, strategies and activities, designed or partly or fully implemented. Evidence of policy implementation took into consideration documents describing initiatives within all the steps of the policy cycle: agenda-setting, policy formulation and development, policy adoption, policy implementation and policy monitoring/evaluation. The focus was on policies in place during the policy mapping exercise (September 2022-December 2023).

 To capture specific contexts, each country team (composed of three members per team) was in charge of conducting a desk review of relevant policy documents, mainly grey literature, including national and local policy documents, plans, strategies, guidelines and legislation, both in paper or virtual form, and any other related reports by national and local governments or by non-governmental organizations (NGOs) related to the 44 proposed policies. Supplementary file 1 contains additional information on the country teams (AD, KKP, OMM, SKA, RRAY, BA, MMG, LL, PN, JSM, CM, CKN, RS, CS, PMDDS, and TS). The documents were searched online on the official websites of the respective governments, and through official requests to the national and local ministries. A scope document containing definitions and aspects to be included or excluded for each policy guided the completion of the template. Box 1 outlines the template that guided the data collection to be completed by the researchers of each country team for the policy mapping, per policy document.

**Box 1. Predetermined Template to Compile and Extract the Data Gathered From the Policy Mapping**

**Title of Proposed Policy**

[Proposed policy]

**What is the context for this policy in your country?**

(Please explain anything related to this policy that does not count as evidence but is still relevant to know)

**Is this policy applicable to your country? **Yes/No

If “No,” please specify the reason:

*[If this policy is not applicable, leave the information below blank and move to the next one]*

**Level of jurisdiction:** Local/Regional/National/Shared jurisdiction/Other

If “Shared jurisdiction” or “Other,” please specify: ___________

**Evidence of policy implementation**

**Type of policy:** legislation/strategy/plan/guideline/intention/funding/other:

(Please specify:_________)

**Has it been approved/implemented?** Yes (date:____)/No

**Name of policy:**

**Aim(s):**

**Summary of the policy:**

(Please describe in 1 paragraph how the policy covers the information from the proposed policy)

**Inequalities/vulnerable groups:**

(Please explain if the evidence takes into account nutrition-related inequalities or vulnerable groups)

**Gender inequalities/women’s empowerment:**

(Please explain if the evidence takes into account gender equality or women’s empowerment)

**Monitoring/enforcement:**

(Please explain any type of monitoring/enforcement mechanisms related to this policy)

**Is this policy part of any overarching plan(s) by the government?** Yes (Please specify: ___________) / No

**Observations/additional comments:**

The data sources analysed included national and local-level strategy documents (from each selected city), corresponding action plans (such as national food and nutrition security plans, national policies for the prevention of diet-related NCDs, and national plans to reduce climate change and/or biodiversity loss), single policies, actions or guidelines and national funding mechanisms and grants for projects related to the implementation of these policies. All retrieved documents were analysed to describe all the food system policies in the countries and cities studied, and specific aspects that the policies address (eg, type of outcome, social aspects considered).

 The exploratory policy mapping was carried out ahead of the interviews to inform the focus of the interviews with stakeholders. These policy mapping results were also used during the iterative thematic analysis process.

####  Policy Stakeholder Interviews

 We sought to interview stakeholders involved in agriculture, trade, environment, health, consumer rights, nutrition and food policy issues at national or local levels. The stakeholders had to have experience in the context of the development or implementation of food system policies, or to have played a role in the policy cycle process in their country or city, and had to be employed in one of the following organisation categories: (*i*) national or local government, (*ii*) international organisation or national NGO, and (*iii*) academia or research institute within the country or city. Stakeholders from the private sector were not included given their role within commercial determinants of health in the policy process.^24-26^

 The stakeholders were identified using a sampling technique in two stages. The first stage was done by each country team. A list of stakeholders was compiled based on publicly available data sources (including government websites, scientific publications, NGO publications, and the Internet) or from previous projects that involved engagement with policy-makers, NGOs, other international organisations, or academia. The second stage was a pseudo-anonymised screening (without any sort of identification details) by two independent researchers (CB and IVD). The criteria for determining the number and types of participants interviewed was based on securing sufficient diverse characteristics (in terms of gender – when possible, field of expertise, organisation type) across the five countries and cities included in the study. Forty stakeholders were selected (eight per country, four at national and four at local level), and were assigned two additional replacement options with similar expertise in case they would not be available for an interview or failed to respond.

 An interview guide containing key themes across multiple contexts and levels of influence was initially developed, based on a review of the literature conducting similar interviews on barriers and facilitators to policy development and implementation across different settings and countries^27-34^ and using the Health Policy Triangle (HPT) framework, as it has been extensively used at local, national and regional levels to assess health policies^35^ and its use in low- and middle-income countries has been reported.^36^ A copy of the final interview guide used is provided in Supplementary file 2.

 Forty stakeholders were initially selected and invited to participate in the study. A formal invitation letter, an informed written consent and an overview of the project were sent to them. The stakeholders who agreed to participate were requested to confirm their participation via email. With no responses from the stakeholders, one email was sent as a reminder. If the situation persisted or the stakeholder rejected to participate, their replacement option was contacted. Out of the forty stakeholders initially selected, two did not answer and three were not available and suggested a colleague to replace them.

 Data were collected using semi-structured interviews. Based on the study protocol, stakeholders were asked to give their views and reflect on their professional experiences with the design and/or implementation process for food system policies in their country/city. Each interview was carried out in French or English, and was recorded and transcribed by national researchers involved in the policy mapping exercise. The interview was finalised when no new information was being provided (ie, data saturation was reached). The interviews were conducted between May and October 2023 and lasted approximately 60-90 minutes. Interviews were imported into NVivo 14 software to be analysed.

 To answer the research question—what are the main barriers and facilitators that governments face when attempting to design and implement food system policies?—the interview data were analysed using thematic framework analysis based on Boyatzis’ hybrid approach, as it allows to identify associations between themes and put forward propositions,^37^ by constructing a conceptual model of their findings through a series of steps, including keyword and quotation selection, coding, theming, interpretation. This approach blends data-driven, inductive identification of themes in the data with a research-driven, deductive approach using prior research as a guide for articulating meaningful themes.^37^ The code scheme was informed by the HPT framework, as it outlines four key intersecting elements of healthy policy-making: the actors, the context, the content and the process. This framework was chosen as it was composed of the core aspects relevant to the study of global health and food policies, and has been used by authors in similar research.^38-40^

## Results

###  Policy Mapping

 For each country, 40 to 60 documents were included for review (Supplementary file 3). The 44 proposed policies within the Food Systems Policy index were used to describe the number of current policies designed or implemented across the countries and cities, as well as to highlight the main gaps (Table 1).

**Table 1:** The Number of Policies Designed or Implemented and the Main Policy Gaps Related to the Subdomains Within the Food Systems Policy Index, Across the African Countries and Cities Studied

		**National Level**					**City Level**				
		Benin	Cote d'Ivoire	Senegal	Togo	Uganda	Ouidah	Bouaké	Saint-Louis	Sokodé	Mbale
Production	Sustainable carbon sequestration practices	3	2	3	2	1	0	0	0	0	0
	Sustainable fisheries	3	2	6	1	3	0	0	1	0	0
	Regenerative agriculture	2	1	4	2	1	0	0	0	1	0
	Optimisation of water resources management	3	1	3	2	4	0	0	1	0	1
	Incentives for crop, fish and livestock diversification	0	0	2	0	1	0	0	0	1	0
	Evidence-based use of bio-fortification programmes	2	0	2	1	3	0	0	0	0	0
	Land use management	2	2	1	0	2	0	0	1	0	1
	Reduce the use of fertilizers	0	0	0	0	0	0	0	0	1	1
	Reduce the use of pesticides	0	0	0	1	0	0	0	0	0	0
	Subsidies for sustainable healthy crops/livestock/fish	1	1	1	0	1	1	1	0	0	1
	Farmers access to traditional seeds and breeds	2	0	1	2	4	0	0	0	0	0
	Farmers and fishers' support	5	2	5	2	3	0	1	1	0	0
	Support to women's empowerment	1	0	1	0	4	0	0	1	0	0
	Support to young generations	1	2	1	2	4	0	0	1	1	0
	Ecosystem restoration and conservation	2	3	5	5	4	0	0	1	0	0
	Climate change impact preparedness	2	1	3	3	2	0	0	0	0	0
Storage processing packaging distribution	Connecting smallholder farmers with territorial markets	5	2	4	1	2	0	1	0	1	0
	Support for startups and SMEs producing more sustainable and healthier foods	0	1	0	1	0	0	0	0	0	0
	Evidence-based use of fortification programmes	3	1	2	2	1	0	0	0	0	0
	Environmental impact measures	0	0	0	0	0	0	0	0	0	0
Loss & waste	Food loss prevention and reduction through infrastructure investment	0	1	2	1	2	0	0	0	0	0
	Food loss and waste reduction through a step-wise process	0	0	0	0	0	0	0	0	0	0
	Regulation framework at retail level	0	1	0	0	0	0	0	0	0	0
Trade	Risk impact assessment of trade and investment agreements	0	1	0	1	3	0	0	0	0	0
	Effective use of trade policy levers for sustainable food systems	0	0	0	0	0	0	0	0	0	0
	Trade incentives for shorter food supply chains	0	0	0	1	0	0	0	0	0	0
	Transparency of global food supply chains	0	0	0	0	0	0	0	0	0	0
Composition	Reformulation of processed foods	0	0	1	0	0	0	0	0	0	0
	Reformulation of out-of-home meals	0	0	0	0	0	0	0	0	0	0
Labelling	Nutrition information panels and ingredient lists	1	2	1	0	2	0	0	0	0	0
	Evidence-based claim regulations	2	2	1	0	3	0	0	0	0	0
	Front-of-pack labelling	0	0	0	0	0	0	0	0	0	0
	Out-of-home eating outlets menu labelling	0	0	0	0	0	0	0	0	0	0
Promotion	Marketing restrictions of less healthy and less sustainable foods to children across all media	1	0	1	1	1	0	0	0	0	0
	Restriction of marketing of less healthy and less sustainable food in retail outlets	0	0	0	0	0	0	0	0	0	0
	Marketing restrictions of breastmilk substitutes	1	1	1	0	2	0	0	0	0	0
Provision	School food and nutrition policies	2	3	3	5	4	0	0	0	0	0
	Public sector setting (other than school) food and nutrition policies	0	0	0	2	0	0	0	0	0	0
	Support for shorter food supply chains	1	1	2	1	2	0	0	1	0	0
Retail	Zoning laws	0	0	0	0	0	0	0	0	0	0
	Prominence of healthier, more sustainable foods in the (in)formal food sector	0	0	0	0	0	0	0	0	0	0
Prices	Taxes on less healthy, less sustainable foods	1	0	0	1	1	0	0	0	0	0
	Subsidies for healthier and more sustainable foods	1	0	1	1	1	0	0	0	0	0
	Affordability of healthier and more sustainable diets	1	2	3	1	0	0	0	0	0	0
	**Total number of relevant policies covered**	**48**	**35**	**60**	**42**	**61**	**1**	**3**	**8**	**5**	**4**

Abbreviation: SMEs, small- and medium-sized enterprises.

 Based on the documents reviewed, the main food systems policy priorities per country and city could be derived. In all countries, the main priorities were related to the policy subdomains of “food production” (n = 144), “food storage, processing, packaging and distribution” (n = 25), “food provision” (n = 26) and “food prices” (n = 14), generally under the direction of the ministries of agriculture or health. The policy subdomains with the lowest coverage across countries were “food composition” (n = 1) and “food retail” (n = 0). At city level, only very few policies addressing food systems were found and were mainly related to the “food production” subdomain.

 In Benin, no overarching policy strategies with multiple policy actions at the national level were found. In the rest of the countries, several documents collected were part of national, overarching policy strategies. For instance, Côte d’Ivoire’s National Plan of Development (2021-2025) focuses on both population nutrition and climate change, among other social and economic challenges. Senegal counts on four different overarching plans: the Emergent Senegal Plan (PSE 2014–2035) and the National Strategy for Sustainable Development, both of which have a strong emphasis on undernutrition and climate change; the National Plan of Actions for the Environment focusing on climate change; and the National Policy for Nutrition Development which focuses on actions to prevent undernutrition and obesity. Togo’s National Plan of Sanitary Development also focuses on undernutrition and obesity, among other healthcare-related challenges, and the National Plan of Development for Togo includes specific actions focusing on undernutrition and climate change, among others. Lastly, Uganda’s National Development Plan is an overarching policy strategy which includes different policy actions to address undernutrition and obesity, while the Uganda Vision 2040 Strategy focuses mainly on undernutrition and climate change, among other social and economic challenges.

 Although the overall challenges tackled were similar, ie, nutrition and food insecurity, climate change, across the plans and strategies the countries differed in the type of policies designed or implemented. Namely individual legislative acts vs. multisectoral strategies with various policy areas. All countries have shown efforts in the implementation of policies to improve environmental sustainability and food security, through agricultural production policies, for example through incentives to connect agricultural production with environmental objectives, to increase the optimisation of natural resources, to use biofortification programmes, to provide financial support to farmers/fishers (including through infrastructure to create rural-urban links, and through support mechanisms to mitigate climate change effects). In addition, Benin, Senegal and Uganda have shown efforts at national level to improve population diets by increasing accessibility to healthier foods, for example through actions to reduce marketing of unhealthy foods and breastmilk substitutes, to support school food programmes, and to implement taxes on some unhealthy foods.

 Regarding policy gaps, there were similarities across countries concerning the lack of actions to reduce synthetic fertilisers and pesticides, to use systems that quantify the environmental impacts of agri-food companies, to reduce food waste at retailers and consumer levels, to incentivise trade agreements that include health and environmental sustainability, to use front-of-pack labels, to implement reformulation strategies and to modify the availability of healthier foods in urban areas.

 However, several country differences in policy gaps were also observed. For instance, Senegal and Uganda have implemented more policies compared to the other countries, showing efforts to tackle food insecurity and climate change simultaneously, mainly within the food supply chains domain by incentivising policies towards regenerative agriculture, the use of diversification methods in agriculture, the financing of forestry projects and ecosystem restoration mechanisms, and investing in infrastructure to reduce food loss. While social aspects such as support for women’s empowerment in agriculture have been considered only in Benin, Senegal and Uganda, the support for small- and medium-sized companies have been more prominent in Côte d’Ivoire and Togo. In regards to improving food environments, Benin, Côte d’Ivoire, Senegal, and Uganda implemented more policies within the areas of food labelling and marketing restrictions of breastmilk substitutes when compared to Togo, which has mainly focused the efforts on fiscal measures and food provision strategies.

###  Best Available Practices Identified for Food Supply Chains

 Illustrative examples of best available practices have been identified. Best available practices are those policies that best correspond to the respective proposed policy statements within the Food Systems Policy Index^20^ and therefore set relevant standards for governments. In this line, Benin has shown efforts to ensure the sustainability of food production through the Framework Law N° 2014-19 of August 7, 2014 on fishing and aquaculture, whose goal is ensuring the sustainability of fisheries for future generations and the protection of the ecosystems, the preservation of the diversity of marine biology and species. Similarly, Côte d’Ivoire has recently implemented the National Strategy for Sustainable Fisheries Management, a national policy for the development of livestock, fisheries and aquaculture, with a strong focus on ensuring food security. Moreover, the country also provides support for the prevention and reduction of post-harvest losses of fish products within the Regulatory Framework from 2014.

 In Senegal, both the National Food Security and Resilience Support Programme and the 2018-2030 Country Programme show the government’s efforts to progress towards nutrition and food security while simultaneously addressing sustainability challenges. These two programmes are multi-sectoral and touch upon different aspects proposed to improve food systems (from agroecology and sustainable agriculture practices to reforestation strategies of the Great Green Wall of the country). Another good-practice example available within the National Food Security and Resilience Support Programme is related to the prevention of food loss, which aims to reduce by 50% the losses associated with agriculture and fisheries, providing infrastructure support for these sectors. Similarly, the Agricultural Programme for Sustainable Food Sovereignty included both infrastructure support and monitoring efforts to tackle postharvest losses.

 Togo’s National Biodiversity Strategy and Action Plan aimed to restore and preserve national terrestrial and aquatic ecosystems to prevent vegetation and biodiversity loss. In Uganda, diverse strategies have been implemented to regulate water management for agriculture, one of them being the Uganda National Irrigation Policy, which aims to implement an efficient use of water for irrigation purposes to ensure agricultural production and productivity.

###  Best Available Practices Identified for Food Environments

 Côte d’Ivoire has implemented the Decree n° 2013-416 of June 6, 2013 to regulate the marketing of breast-milk substitutes, which is monitored and evaluated by the Ministry of Health. This Decree explicitly includes strict regulations regarding the promotion, packaging and labelling of infant formulas.

 Through the Multi-sector Nutrition Strategic Plan (2018-2022), Senegal was the only country studied that has encouraged the reformulation of processed food products to reduce the content of sugar, fat and sodium, as part of an overall strategy from the government to prevent chronic malnutrition, acute malnutrition, obesity and NCDs.

###  Policy Stakeholder Interviews

####  Stakeholder Characteristics

 Out of the 40 interviews conducted, 3 of them were excluded as their quality was deemed insufficient and did not provide information related to the research question. The detailed reasons for exclusion are available in Supplementary file 4. The full characteristics of the 37 interviewees are available in Table 2.

**Table 2:** Summary of Interviewed Stakeholders and Their Characteristics

**Setting and Number of Interviews Included**		**Organisation**			**Gender**		**Food Systems Policy Domain**		
		**GO**	**NGO**	**AC**	**Male**	**Female**	**FSC**	**FE**	**Both**
Benin	4	3	1	-	4	-	4	-	-
Ouidah	4	4	-	-	4	-	3	1	-
Côte d’Ivoire	3	2	1	-	2	1	-	3	-
Bouaké	3	3*	-	1	3*	1	1	2	-
Senegal	4	2	2	-	-	4	1	2	1
Saint-Louis	4	3	1	-	3	1	3	1	-
Togo	4	2	2	-	4	-	3	1	-
Sokodé	4	4	-	-	3	1	3	1	-
Uganda	4	1	2	1	2	2	1	3	-
Mbale	3	1	1	1	2	1	2	1	-
Total	37	25	10	3	27	11	21	15	1

Abbreviations: GO, government; NGO, non-governmental organization; AC, academia; FSC, food supply chains; FE, food environments. * Two stakeholders were present during one of the interviews in Bouaké.

####  Key Themes

 A total of 21 themes and 90 subthemes arose from previous literature and the analysis of the interview data. The elements derived were organised within the four pre-specified categories of the HPT framework (actors, context, content and process) (Supplementary file 4). In total, 74 barriers and 73 facilitators were identified from the analysis of the interview data.

####  Overall Findings

 Depending on the circumstance and setting, the same theme could be either identified as a barrier or as a facilitator. However, a few themes were only identified as barriers. This was the case with some factors within the context category, such as being close to the period of political elections, regulations related to land use and planning, and overall poverty in the country/region. Other themes that were identified solely as barriers were the language used for policy and regulatory documents, and clarity aspects regarding the timelines in which policy regulations shall be fully implemented. On the other hand, it also happened that some themes were only identified as facilitators. Within the actors’ category, both religious organisations and trade platforms/labour unions were only associated with enabling factors. Similarly, in the case of the policy process, having a sound scientific basis and research was only perceived as a facilitator.

 Moreover, there were some themes identified during the deductive phase that were not mentioned during the interviews. These were mainly actors (ie, an individual industry/business spokesperson, a celebrity, or Indigenous communities or protest groups), but also specific context themes (ie, religion), or factors related to content (ie, official press releases from the government, or industry pledges and self-regulations).

####  Barriers 

 The most prominent themes that emerged from the policy stakeholder interviews as barriers to the design or implementation of food system policies were:

#####  Actors

 The national government was generally seen as the main barrier across countries. Stakeholders associated lower policy development and implementation by the national governments with other factors seen as barriers, such as lack of political will or lack of funding allocated by the government. Other arguments associated with national governments were the lack of accountability mechanisms and transparency in policy development or during the implementation phases, the absence of individual and organisational leaders and the poor political desire to promote food systems policies.

 “*If we had spearheaded with the environment, maybe the head of state wouldn’t have been interested because, at the end of the day, they would want to see, to what extent does this organic policy contribute to forex [foreign exchange]” *[NGO, FSC, National, Female].

 Other actors also perceived as barriers were farmers/fishers, agri-food companies and industries, and individual politicians, whose individual interests may sometimes be threatened by food system policies.

 “*We were warned that we should never come back here; otherwise, we would be slaughtered from the wetland. Rice growers are very hostile when it comes to issues concerning wetlands” *[GO, FSC, Local, Female].

 The general public was often mentioned when talking about barriers, as it was perceived as an unaware stakeholder of the relevance that food systems policies may have within their everyday life. This was associated often with the absence of social or environmental awareness, low support for the policy process in the media and the community, and social disagreements based on cultural and social beliefs, or even local norms. This aspect was also associated with poor social demands in policy reforms from the community.

#####  Context

 Public opinion and lack of awareness from different groups (ie, politicians, the general public, farmers/fishers) about the impact of food and agriculture on health and the environment were often described as the main barriers. This challenge was also perceived to affect governments’ political decisions to act upon this area. Similarly, political will was mentioned across all groups of stakeholders, who saw it as a strong barrier to the design and implementation of food system policies. The absence of political desire to promote and support food systems policies was mentioned across countries and levels of jurisdiction, highlighting the lack of adequate organisational leaders to pursue policy action in the fields of public health and environmental sustainability.

 “*Producers watch much TV and that is why nowadays they’re not informed about what’s going on around them. They need awareness-raising to show them that if you come here, the State will help you” *[GO, FSC, Local, Male].

 Another challenge noted was the country’s political structure and bureaucratic procedures for designing and implementing policies, usually described as slow, rigid and with many layers that make the decision-making process too slow and difficult. Such difficulties related to administrative policy processes, including conflicting interests, mandates, and even goals, were perceived as a barrier by all stakeholder sectors and across countries.

 “*Another blocking factor is the slowness of the administration, especially with the municipal teams. It is very difficult and the administration of these projects requires a lot of paperwork. [...] There is a lot of slowness on the administrative side” *[NGO, FSC, Local, Male].

 The prominence of food available related to international and exogenous factors, such as international trade agreements with third countries or the presence of multinational companies in the national territory were also mentioned when describing potential barriers to the adoption of policies, mainly by stakeholders working in the government.

 “*When you arrive in villages today, there is no food; everything that can be eaten is imported. And it is industrial (processed) food” *[GO, FSC, Local, Male].

 More generally, the country’s political instability, regional or internal conflicts, international wars and conflicts, and international agreements were also perceived as barriers to the development and implementation of food systems policies.

#####  Content

 Inadequate funding and lack of transparency on how the funding available is prioritised were perceived as the major barriers to the design and implementation of food system policies. It impacted policy at different levels, from the speed of the process to the resources allocated to accomplish the objectives of the programmes. The common absences, or oftentimes insufficiency, of resources related to finance, infrastructure, training, skills or evidence were described by most stakeholders as the main barrier, and it was often linked to a lack of political will and leadership from the government, or to the complexity of the agreements across ministries to allocate budget and resources.

 “*When it reaches the Solicitor General, that is cash. You need something to put there. [...] The local government keeps quiet because there is no budget. That is the main problem given that we don’t have the budget for the formulation of bills” *[GO, FSC, Local, Female].

 To a lesser extent, the challenges associated with the budget were sometimes linked to a lack of human resources, mainly during the implementation process.

 “*Even if the texts are good, who applies them? We don’t have regional management; in fact, we don’t even have any specialists within our own ranks. And yet we have the manpower, but what is being done?” *[GO, FSC, Local, Male].

 Another challenge that raised concern among stakeholders during the development and implementation of policies was the aspect of clarity with the content (ie, scientific data, definitions and terminology, language), with the assignment of coordination and accountability when the responsibilities were shared across ministries or partners, and with implementation dates and the overall sustainability of the policy to be effective and applicable in the future.

 “*Unfortunately, the law on consumer protection states that authorisation must be delegated by the competent bodies, which has yet to be specified. And everyone is pulling their way”* [GO, FSC, National, Female].

#####  Process

 The negotiation process around the design of policies was seen as a challenge by different stakeholders, mainly due to inefficient and biased cross-government and cross-ministries exchanges, and to a lesser degree to advocacy and lobbying actions by the private sector and civil society organisations, and the partnerships created among stakeholders.

 “*What is lacking is good coordination between these institutions and the supervisory ministries, and good collaboration between them at all levels”* [NGO, General, National, Female].

 All groups of stakeholders perceived the implementation process of policies as a main challenge, often described as slow, incomplete or insufficient. This was often linked to the complexity experienced with the bureaucratic system of the administration, and the difficulties associated with the administrative process in cases of conflicting mandates, responsibilities and interests. Moreover, the implementation process was also seen as a contributing barrier to speeding up government actions in food systems.

 “*The texts exist, but it’s the application that’s the problem. [...] Often, many projects and initiatives are developed, but do not achieve the development objectives set out at the outset” *[AC, FSC, Local, Male].

 The absence of effective monitoring and evaluation mechanisms was also perceived by all groups as a contributing barrier, both in enforcing actions and in advancing future similar interventions by the government. The absence of routine monitoring to determine the effectiveness of policies and their compliance was a key barrier to policy implementation, often associated with the lack of mechanisms to hold stakeholders or national/local agencies accountable for policy inaction or partial implementation.

 “*Monitoring, evaluation and accountability are the three fundamental elements. These three elements suffer in current policies” *[NGO, FSC, National, Female].

####  Facilitators 

 The most prominent themes that emerged from the policy stakeholder interviews as facilitators to the design or implementation of food system policies were:

#####  Actors

 All groups of stakeholders identified the national government as the main actor enabling the development and implementation of food system policies. This included primarily the Ministries of Agriculture, Health and Social Affairs, and to a lesser extent the Ministries of Environment and Trade. The positive perception was often associated with leadership characteristics and strong awareness matching with the ambition to promote policies.

 “*Government commitment. If the government is committed and sensitive enough to these results in the short and long term, I think that would facilitate the rest” *[NGO, FSC, National, Male].

 The general public was also considered an important facilitating actor, as it was perceived as a key element that enacted pressure on the government to act and to achieve the success and sustainability of policies over time.

 “*The partner can withdraw, but the beneficiary population must ensure the continuity of the project” *[GO, FSC, Local, Male].

 Another key enabling actor identified were international organisations, as they were perceived to incentivise governments to act through soft pressure and different technical support mechanisms. It was common for all countries to describe influences from international organisations or countries abroad that facilitated the policy process, highlighting aspects such as engagement, collaboration and support, mainly during the policy design and sometimes during the initial stages of the policy implementation processes.

 “*These development agencies that have money can come and do some pilots. If those pilots succeed gradually, we can get the government to buy in when they see that something is working” *[AC, FE, National, Female].

 Public sector agencies were perceived by all stakeholder groups as facilitators of the design and implementation of policies, as they tended to be associated with independent research and as key actors in the evaluation of policies. However, they recognised that they had limited power as they tended to depend on allocated funding or requests for advice from the national governments.

 “*Today, you see, the ministries exist, but when the government opts for agencies, opts for parallel structures to carry out certain activities, this seriously hampers and even kills the ministries, which are very important, and nothing can be done about it” *[GO, FSC, National, Female].

 Other enabling actors identified were national and local NGOs, mainly related to factors associated with social aspects and undernutrition, and community groups that were able to voice and mobilise different sectors of society that influence policy. This aspect was commonly perceived as crucial during the initial phases of the policy cycle, associated with social acceptance, consciousness and awareness of benefits from the community.

 “*The CSOs are Civil Society Organisations, everything that is an NGO, everything that is a consumer association, for example. They have a major role to play in alerting the population and drawing the attention of politicians to what is going wrong on the ground”* [GO, FE, National, Female].

 International governments, including policy actions from, countries with similar approaches, challenges or government structures, were also perceived as enablers if they had implemented similar policies showing promising results, or whose political reputation was deemed high.

 “*So if a product is banned somewhere else, it’s banned here too” *[GO, FSC, National, Male].

 Similarly, actions that quantify, compare or benchmark actions and initiatives by governments from neighbouring countries were perceived as a facilitating factor to incentivise national and local governments to take action.

 “*Benchmarking on neighbouring countries and other countries I think is a big enabler in setting an improved policy environment” *[NGO, FE, National, Male].

#####  Context

 Across countries, stakeholders recognised public opinion and awareness as a facilitating factor to the development and implementation of food system policies. The common arguments around it were their link to government accountability or to pressuring the industry to make changes.

 “*Let these people be held accountable. You see, if you make the community sensitized, they will know their rights and they will be able to demand from their leaders” *[NGO, FE, Local, Female].

 International agreements were also perceived as catalysts for the development and implementation of food system policies. The SDGs were often referred to as an example of international agreements signed that exerted pressure on the government to act within food systems.

 “*All this is covered by the international agreements we have signed [...] We have to respect the various agreements we have signed” *[GO, FSC, Local, Male].

 Stakeholders from all the groups mentioned strong political will as a key enabler and provided some examples of successful cases in which policies were designed and approved because of strong leadership coming from the higher levels of political leadership.

 “*For incentive measures, you first need political will, you need synergy between all the stakeholders, and above all that. […] The State could do better in this area, but it needs real political will, because that is what is really lacking” *[GO, FSC, Local, Male].

#####  Content

 Sufficient financial resources were often perceived as one key facilitator to the development and implementation of food system policies, and it was often associated with a higher level of prioritisation by the government and strong political will. It was also linked to manpower and knowledge translation through formation and education resources.

 “*The strategic plans that flow from the strategies we develop must be financed in order to implement our policies” *[GO, FSC, National, Male].

 Technical resources in the form of scientific data were also highlighted as a key factor in the policy process and were often seen as an indispensable factor linked to the agenda-setting and consultation processes with policy actors.

 “*The evidence. The first thing that facilitates policy development is evidence. This is a key point because the evidence does not lie and allows decision-makers to be much more comfortable in their orientations, but it also allows us, as civil society organisations, to be more comfortable in our support because everything is based on evidence” *[NGO, FSC, National, Female].

 More generally, overall policy objectives were mentioned by some stakeholders as facilitators to the development and implementation of food system policies, in particular when analysing the context of the country and the main challenges already faced now that will worsen.

 “*When the population is sick, what returns can we expect tomorrow?” *[GO, FSC, Local, Male].

#####  Process

 An important theme that emerged from all stakeholder groups was the agenda-setting step, as it was seen as a key determinant of the success of a policy over time. It was therefore associated with strong political will and leadership, but also with data availability and international pressure.

 “*What will encourage or facilitate the use of natural fertilisers is awareness-raising. We need to make the people who pass the laws and the governments aware of the need to move in that direction” *[NGO, FE, National, Male].

 The inclusion of interest groups during the policy consultation process was identified as an enabling factor, recognising these participatory mechanisms among cross-sectoral stakeholders to enhance the effectiveness and democratisation of the process. However, some argued that this type of consultation had limited power to drive the policy into action if the political will was lacking or the interests of some powerful actors (ie, the food industry) were prioritised over others.

*“In this country, we are committed to working with stakeholders to achieve the indicators. The dialogue on food systems has given people a better understanding of the difficulties” *[AC, FE, National, Female].

 Effective negotiation (ie, advocacy, cross-governmental collaboration and multi-stakeholder platforms) were mentioned as key facilitators. Stakeholders recognised the importance of participatory mechanisms to enhance process’ effectiveness but recognised the power imbalances between actors involved in multi-stakeholder partnership.

 “*Food policies can only be improved when all stakeholders are involved. You have to involve everyone, whether they are opposed to the policy or not. [...] It is by involving everyone that we can have an effective food policy” *[NGO, FE, National, Male].

 Among all stakeholder groups, there was a perception that effective monitoring and evaluation mechanisms are essential factors that can facilitate the design and implementation of future policies. Stakeholders noted that if there are successful cases of policies being enforced that work, governments will be more willing to implement future regulations.

## Discussion

 In this study, we analysed the type and scope of food system policies designed or implemented by national and local governments in five selected countries. Then, the experiences and perceptions of barriers and facilitators when designing and implementing food system policies were critically analysed. The rationale for this research was to provide insights that inform future food systems policy design and implementation processes in national and local jurisdictions across the African region and potentially other low- and middle-income countries.

 The identified policies during the policy mapping cut across diverse food system domains: there were production strategies for improved dietary diversity, regulations of food environment factors (which involved mainly actions to increase the affordability of healthier products, marketing restrictions to children and, in some countries, nutrition labelling), and strategies for nudging dietary habits into healthier ones (which consisted mainly on school food programmes). Many policy gaps remain both at national and local levels across all the subdomains studied. Particularly, policies in the areas of food loss and waste, food trade, food composition and food retail were missing across countries.

 The results from the policy mapping showed that the five African countries are designing and implementing policies that simultaneously address food insecurity and climate change, mainly focusing on food production. This may be due to the central role that agriculture/fisheries play in food security, to the fact that climate change significantly contributes to the challenges associated with food insecurity in SSA,^41^ but also considering that agriculture has been in the political and scientific debates over the last decade concerning its impacts on greenhouse gas emissions^42^ and its change needed to transform food systems.^43^ However, the focus on food security discourses within the field of food production seems to have remained pronounced in the studied countries. This may be because political discourse seems to be dominated by the belief that food security shall be centrally addressed by producing more food.^44^ Despite the rise in NCDs, government policies seem to continue emphasising agricultural production of staple commodities while supporting the food industry, motivated by conventional perspectives on food security, economics and trade agreements.^45^ Another reason may be the high percentage of employment in agriculture across the countries studied, and therefore economic value generated for the internal market. While the focus on food insecurity through agriculture remains very relevant, not enough attention has been paid in the five African countries studied to achieve non-obesogenic food supply chains, as the results from the policy mapping show that in the domains of agriculture, food production and processing the healthiness of population diets were not considered.

 Similarly, within food environments, the countries studied are starting to design and implement some policies focusing on obesity prevention, mainly for the subdomains of food provision and food prices. Nevertheless, the focus of food environments seems to target mainly undernutrition and food security aspects, and the overall results show that food system-related interventions are not yet obesity-specific. Instead, they tap into the wider field of healthy and nutritious diets and therefore sometimes emerge within policy strategies to create non-obesogenic food environments, but it did not seem to be a focus to create non-obesogenic food supply chains. Yet, within food supply chains the focus should also be on improving nutrition and population health, considering malnutrition in all its forms (including not only food security and undernutrition but also micronutrient deficiencies, overweight and obesity)^46^ and environmental degradation aspects, in line with the Global Syndemic. An underlying potential explanation regarding obesity may be cultural, as in some developing countries childhood overweight is associated with wealth, and the consumption of unhealthy foods is a way of showing socio-economic status.^47^

 Regardless of the jurisdictional context or the geographical region, policy stakeholders experienced common political and technical issues when talking about the design and implementation process of food system policies.

 The national government was often mentioned as a main barrier, especially in cases of fragmented levels of government (with overlapping jurisdictions at federal, national, and local) that can threaten the effective design and implementation of food systems policies. This is, however, understandable considering that the government acts taking into account different views and values across ministries with varied political agendas, and food-related issues are not necessarily central and aligned across ministries’ missions.

 The major barriers to policy development and implementation perceived by all groups of stakeholders were insufficient financial resources, lack of political will, limited data and lack of implementation, and inadequate monitoring and enforcement mechanisms. These results are in line with previously conducted research on the barriers and facilitators towards the development of food-related policies.^29,31,38,48^ From an implementation perspective, these barriers could be tackled by increasing awareness across all the stakeholders involved, producing more scientific data that is context-specific for the country/city, and ensuring strong leadership support throughout the whole process. Moreover, a better understanding is needed as to why some stakeholders may have negative perceptions of food systems policies, before making efforts towards their implementation. Lastly, involvement from multiple stakeholders is likely to be key in achieving the proper design and implementation of food system policies. While dealing with power dynamics is a delicate process, potential ways to achieve successful multi-stakeholder engagements are independent mediation based on trust and transparency, and visualising and openly discussing the hidden power dynamics identified in the group.^49^

 As mentioned during a few interviews, corruption was also identified as another aspect of relevance that acts as a barrier during the policy implementation process, undermining good governance and trust in the political institutions. This complex multi-sector phenomenon is prominent globally across private and public sectors. In the data analysed, the forms of corruption mentioned included bribery, ties of families and tribes, and lobbying. Even if these aspects were described superficially while describing lived experiences during the implementation of policies with private actors or elected officials and politicians, it highlights the need to tackle these practices during the policy process.

 The most commonly perceived facilitators by all groups were supportive public opinion and population awareness, international agreements and commitment signed, sound agenda-setting and multi-sector and multi-stakeholder consultations and partnerships, availability of financial resources and data, and solid political will. Similarly, these results also go in line with similar food-related policy studies^28,29,38,40,45^ that highlight the relevance of leadership and political dialogues, accessibility of financial and data resources, and supportive public opinion from citizens. To a lesser extent, programmes implemented with the help of development agencies and international organisations were perceived by both national and local stakeholders to have positive stimulating effects, as they act as starting points that can incentivise further local policy action.

 The main strength of this study is that it informs researchers and governments working on food systems dossiers to identify different potential barriers and facilitators they may face during the policy cycle, with a main focus on the context of five African countries. To the best of our knowledge, this is the first study identifying food system policies developed and/or implemented and the perceived barriers and facilitators for food system policies in African countries. Another key strength of this study is its consideration of the entire food system, from food production to food prices. Moreover, this study used a combination of methods that allowed for the triangulation of the data, to improve the validity of the information obtained during the policy mapping process. Another strength of this study is the fact that data was collected by local researchers, allowing precise analysis of the context and more transparent communication with local policy stakeholders. And that the local researchers conducting the policy mapping were the same ones that conducted the stakeholder interviews, which allowed a clearer understanding of the policy technicalities discussed. This also facilitated the setting of the interviews, as local researchers had been already in touch with policy stakeholders during the policy mapping phase, allowing for high response rates for interview appointments. The fact that the analyses were run by scientists from outside the countries may be an additional strength, as there were no biases towards a specific country or city. Similarly, there was no bias in the analysis of the results concerning the stakeholders interviewed, as they all had a code that did not allow demographic information to be identified. However, this may also be seen as a limiting factor, as some specifications regarding the culture or context may have been overlooked or misinterpreted. Similarly, the multi-partner involvement made the study more challenging, as the food system policies and the interview guide were proposed by different researchers than the ones that conducted the policy mapping and the stakeholder interviews. However, through detailed protocols and information documents and training sessions, we tried to minimise this problem. Across the countries and cities studied, a common problem was the availability of policy data in digital format. Data accessibility posed several constraints, and some of the information included in this research was only available in physical format. Nonetheless, the country teams were provided by the respective ministries with all the documents requested, both digital and physical. The fact that the information was often only available in physical format does not allow us to conclude if the lack of policy documents for some of the policies proposed implies a lack of action by governments (for instance, in food waste or food retail). Another important limitation was the difficulty of finding local researchers who had expertise in food systems. Most of the researchers involved in the data collection process were experts on public health, nutrition or food security. A potential limitation in this regard may be the interpretation of the policy documents gathered, given the legislative nature of the policy documents. This also means that some factors related to environmental sustainability, climate change or social inequalities may have been under-considered.

 The policy mapping results show that the five African countries studied are designing and implementing strategies in line with their international pledges and working on solutions towards sustainable food systems. However, the efforts do not seem to be targeting equally all forms of malnutrition, such as the promotion of healthy diets or the achievement of non-obesogenic food environments, as well as some climate change targets. More action is needed at the policy level to address the Global Syndemic, in particular at local levels of jurisdiction. Additional learnings from this study are that there may be as many facilitating factors as barriers when governments aim to design and implement policies. As seen, technical factors such as adequate financial resources, scientific data and adequate monitoring and enforcement mechanisms play a key role in the success of the policy. Political factors such as political will, government accountability mechanisms, multi-sector partnerships and international agreements seem to be particularly relevant to the success of food system policies. In that line, the analysis of barriers and facilitators provides a reminder to both researchers and policy-makers that designing and implementing food system policies can be more complex than it seems, as there are factors that may escape the control of governments that can either positively or negatively impact the success of the policy. For instance, when it comes to food production policies, governments should focus on more than just ensuring food security and should take into consideration other factors such as environmental sustainability and the diversity and variety of population diets to prevent NCDs.

 Despite the different political contexts and actors involved in this study, several commonalities were perceived. However, the underlying reason behind these challenges may vary per country and region. The data collected did not allow us to explore further the reasons and mechanisms behind some of these barriers that exist across the selected countries (ie, lack of political will, insufficient financial resources, poor monitoring and evaluation mechanisms). These aspects and the underlying roots of the barriers identified in this study should be further explored. Based on these results, future research could also explore the effectiveness of current policies designed and implemented from a systems approach towards the sustainability of food systems. Given the barriers and facilitators identified in this study, researchers could explore ways in which these perceived barriers could be overcome or by modelling potential scenarios that may address them. Using participatory methods with stakeholders, complex system dynamics and building on existing research,^50-52^ future research could contribute further to the existing literature on specific factors that have helped the development of food systems interventions and identify potential ways to overcome the technical and political barriers. For governments, these results can be used as a guide during the policy process, to map and identify potential factors that may help or hamper the development/implementation of food system policies within their country/city. Furthermore, it can be particularly relevant to recognise factors that play an important role in the process to ensure the support and success of policies over time.

## Conclusion

 This article describes a wide range of policies on diverse food systems domains that have been designed and implemented by national and local governments in Benin, Côte d’Ivoire, Senegal, Togo and Uganda, and five of their cities (Ouidah, Bouaké, Saint-Louis, Sokodé, and Mbale) towards healthy and environmentally sustainable food systems. It also explores barriers and facilitating factors to their development and implementation. The research shows that designing and implementing food system policies to address the Global Syndemic can be technically and politically challenging, in particular with regards to legislative, financial and practical aspects. Several common barriers and facilitators were identified despite the different political contexts and actors involved in this study. Understanding the technical and political challenges faced by governments to create healthy and sustainable food systems may contribute to capacity building in their regulatory space.

## Acknowledgements

 The corresponding author would like to thank Eugénie Stoclet for the training and assistance during the analyses conducted in NVivo14.

## Ethical issues

 The full details of the ethical committees from each country that approved this study are avail-able in Supplementary file 1.

## Conflicts of interest

 Authors declare that they have no conflicts of interest.

## Data availability statement

 All the data presented in this study are available upon reasonable request from the corresponding author.

## Supplementary files


Supplementary file 1. Definition of Key Terms and Ethic Approvals.

Supplementary file 2. Interview Guide.

Supplementary file 3. List of Documents Provided by Each Country.

Supplementary file 4. Thematic Codes Identified During the Analysis of the Interviews, and Reasons for Exclusion of Interviews.

## References

[1] Araújo RG, Chavez-Santoscoy RA, Parra-Saldívar R, Melchor-Martínez EM, Iqbal HM (2023). Agro-food systems and environment: sustaining the unsustainable. CurrOpin Environ Sci Health.

[2] Willett W, Rockström J, Loken B (2019). Food in the Anthropocene: the EAT-Lancet Commission on healthy diets from sustainable food systems. Lancet.

[3] Campbell BM, Hansen J, Rioux J, Stirling CM, Twomlow S, Wollenberg E (2018). Urgent action to combat climate change and its impacts (SDG 13): transforming agriculture and food systems. CurrOpin Environ Sustain.

[4] Swinburn BA, Kraak VI, Allender S (2019). The global syndemic of obesity, undernutrition, and climate change: the Lancet Commission report. Lancet.

[5] World Health Organization (WHO). UN Report: Global Hunger Numbers Rose to As Many As 828 Million in 2021. WHO; 2021. https://www.who.int/news/item/06-07-2022-un-report--global-hunger-numbers-rose-to-as-many-as-828-million-in-2021. Accessed October 11, 2024.

[6] World Obesity Federation. World Obesity Atlas 2023. World Obesity Federation; 2023. https://www.worldobesity.org/resources/resource-library/world-obesity-atlas-2023. Accessed October 11, 2024.

[7] Tiseo I. Statista: Global GHG Emission Shares by Sector. Statista; 2023. https://www.statista.com/statistics/241756/proportion-of-energy-in-global-greenhouse-gas-emissions/. Accessed October 11, 2024.

[8] Candel JJ, Biesbroek R (2018). Policy integration in the EU governance of global food security. Food Secur.

[9] Lang T, Mason P (2018). Sustainable diet policy development: implications of multi-criteria and other approaches, 2008-2017. Proc Nutr Soc.

[10] FAO, IFAD, UNICEF, WFP, WHO. The State of Food Security and Nutrition in the World 2020: Transforming Food Systems for Affordable Healthy Diets. 2020. https://www.fao.org/3/ca9692en/online/ca9692en.html. Accessed January 26, 2024.

[11] Swinburn B, Kraak V, Rutter H (2015). Strengthening of accountability systems to create healthy food environments and reduce global obesity. Lancet.

[12] Burgaz C, Gorasso V, Achten WMJ (2023). The effectiveness of food system policies to improve nutrition, nutrition-related inequalities and environmental sustainability: a scoping review. Food Secur.

[13] Aborode AT, Favour Obianuju A, Onyeaka H (2023). Editorial: Obesity and nutrition in the most remote parts of Africa. Front Public Health.

[14] FAO, AUC, ECA, WFP. Africa - Regional Overview of Food Security and Nutrition 2023: Statistics and Trends. Accra, Ghana: FAO, AUC, ECA, WFP; 2023. 10.4060/cc8743en.

[15] Global Network Against Food Crises (GNAFC). Global Report on Food Crises 2022 Mid-Year Update. GNAFC; 2022. https://reliefweb.int/report/world/global-report-food-crises-2022-mid-year-update. Accessed January 26, 2024.

[16] Food and Agriculture Organization (FAO). Transforming the World through Food and Agriculture: FAO and the 2030 Agenda for Sustainable Development. FAO; 2019. https://www.fao.org/documents/card/en/c/CA5299EN/. Accessed January 26, 2024.

[17] United Nations Statistics Division (UNSD). The Sustainable Development Goals Report 2024. UNSD; 2024. https://unstats.un.org/sdgs/report/2024/. Accessed October 7, 2024.

[18] Laar A, Barnes A, Aryeetey R (2020). Implementation of healthy food environment policies to prevent nutrition-related non-communicable diseases in Ghana: national experts’ assessment of government action. Food Policy.

[19] Holdsworth M, Kimenju S, Hallen G, Laar A, Oti SO (2023). Review of policy action for healthy environmentally sustainable food systems in sub-Saharan Africa. CurrOpin Environ Sustain.

[20] Burgaz C, Van-Dam I, Garton K (2024). Which government policies to create sustainable food systems have the potential to simultaneously address undernutrition, obesity and environmental sustainability?. Global Health.

[21] International Development Research Centre (IDRC). Harmonized Indicators for Measuring Progress Toward More Sustainable, Healthier Food Systems. https://idrc-crdi.ca/en/project/harmonized-indicators-measuring-progress-toward-more-sustainable-healthier-food-systems. Accessed April 2, 2024.

[22] Dokua SasuD. Africa: Prevalence of Undernourishment by Country. Statista; 2024. https://www.statista.com/statistics/1305636/prevalence-of-undernourishment-in-africa-by-country/. Accessed October 11, 2024.

[23] World Bank Group. World Bank Open Data. https://data.worldbank.org. Accessed August 26, 2024.

[24] Mialon M (2020). An overview of the commercial determinants of health. Global Health.

[25] Miller D, Harkins C (2010). Corporate strategy, corporate capture: food and alcohol industry lobbying and public health. Crit Soc Policy.

[26] Brownell KD, Warner KE (2009). The perils of ignoring history: Big Tobacco played dirty and millions died. How similar is Big Food? Milbank Q.

[27] Plested BA, Edwards RW, Jumper-Thurman P. Community Readiness: A Handbook for Successful Change. Fort Collins, CO: Tri-Ethnic Center for Prevention Research; 2006.

[28] Rose N, Reeve B, Charlton K (2022). Barriers and enablers for healthy food systems and environments: the role of local governments. CurrNutr Rep.

[29] Phulkerd S, Sacks G, Vandevijvere S, Worsley A, Lawrence M (2017). Barriers and potential facilitators to the implementation of government policies on front-of-pack food labeling and restriction of unhealthy food advertising in Thailand. Food Policy.

[30] Mohamed SF, Juma P, Asiki G, Kyobutungi C (2018). Facilitators and barriers in the formulation and implementation of tobacco control policies in Kenya: a qualitative study. BMC Public Health.

[31] Clarke B, Kwon J, Swinburn B, Sacks G (2021). Understanding the dynamics of obesity prevention policy decision-making using a systems perspective: a case study of Healthy Together Victoria. PLoS One.

[32] Gittell R, Magnusson M, Merenda M. The Sustainable Business Case Book. Saylor Foundation; 2012. https://open.umn.edu/opentextbooks/textbooks/142. Accessed March 21, 2024.

[33] Meiksin R, Er V, Thompson C (2022). Restricting the advertising of high fat, salt and sugar foods on the transport for London estate: process and implementation study. Soc Sci Med.

[34] Kallio H, Pietilä AM, Johnson M, Kangasniemi M (2016). Systematic methodological review: developing a framework for a qualitative semi-structured interview guide. J Adv Nurs.

[35] Zahidie A, Asif S, Iqbal M (2023). Building on the health policy analysis triangle: elucidation of the elements. Pak J Med Sci.

[36] O’Brien GL, Sinnott SJ, Walshe V, Mulcahy M, Byrne S (2020). Health policy triangle framework: narrative review of the recent literature. Health Policy Open.

[37] Boyatzis RE. Transforming Qualitative Information: Thematic Analysis and Code Development. SAGE Publications; 1998.

[38] Sing F, Carriedo A, Mackay S, Tenbensel T, Swinburn B (2023). Barriers and enablers in designing regulations to restrict the exposure of children to unhealthy food and beverage marketing. Front Polit Sci.

[39] Saito J, Keosada N, Tomokawa S (2015). Factors influencing the National School Health Policy implementation in Lao PDR: a multi-level case study. Health Promot Int.

[40] Reeve E, Thow AM, Bell C (2018). Implementation lessons for school food policies and marketing restrictions in the Philippines: a qualitative policy analysis. Global Health.

[41] Fagbemi F, Oke DF, Fajingbesi A (2023). Climate-resilient development: an approach to sustainable food production in sub-Saharan Africa. Future Foods.

[42] Crippa M, Solazzo E, Guizzardi D, Monforti-Ferrario F, Tubiello FN, Leip A (2021). Food systems are responsible for a third of global anthropogenic GHG emissions. Nat Food.

[43] Rivera-Ferre MG (2020). From agriculture to food systems in the IPCC. Glob Chang Biol.

[44] Resnick D, Swinnen J. The Political Economy of Food System Transformation: Pathways to Progress in a Polarized World. Oxford, UK: Oxford University Press; 2023. 10.1093/oso/9780198882121.001.0001.

[45] Mozaffarian D, Angell SY, Lang T, Rivera JA (2018). Role of government policy in nutrition-barriers to and opportunities for healthier eating. BMJ.

[46] Fanzo J, Hunter D, Borelli T, Mattei F. Diversifying Food and Diets: Using Agricultural Biodiversity to Improve Nutrition and Health. Routledge; 2013.

[47] Herens M, ten Hove H, Bakker S, et al. Addressing Overweight and Obesity in LMICs in the Realm of Rural Development and Food Systems: Global Evidence Base versus Practice in Five Country Examples. International Fund for Agricultural Development; 2023.

[48] Rose N, Reeve B, Charlton K (2022). Barriers and enablers for healthy food systems and environments: the role of local governments. CurrNutr Rep.

[49] Brouwer H, Hiemstra W, van Vugt S, Walters H (2013). Analysing stakeholder power dynamics in multi-stakeholder processes: insights of practice from Africa and Asia. KnowlManag Dev J.

[50] Milsom P, Tomoaia-Cotisel A, Smith R, Modisenyane SM, Walls H (2023). Using system dynamics to understand transnational corporate power in diet-related non-communicable disease prevention policy-making: a case study of South Africa. Int J Health Policy Manag.

[51] Mui Y, Ballard E, Lopatin E, Thornton RLJ, Pollack Porter KM, Gittelsohn J (2019). A community-based system dynamics approach suggests solutions for improving healthy food access in a low-income urban environment. PLoS One.

[52] Quinteros-Reyes C, Seferidi P, Guzman-Abello L, Millett C, Bernabé-Ortiz A, Ballard E (2024). Mapping food system drivers of the double burden of malnutrition using community-based system dynamics: a case study in Peru. BMC Glob Public Health.

