# Supplementary material for: Barriers and Facilitators to the Development and Implementation of Public Policies Addressing Food Systems in Five Sub-Saharan African Countries and Five of Their Cities
Source: Int J Health Policy Manag. 2025 Mar 18;14:8592. doi: 10.34172/ijhpm.8592 (PMC12089831; doi:10.34172/ijhpm.8592)
Supplement: Supplementary file 1 — Definition of Key Terms and Ethic Approvals. [file ijhpm-14-8592-s001.pdf]

Article title: Barriers and Facilitators to the Development and Implementation of Public Policies Addressing Food Systems in Five Sub-Saharan African Countries and Five of Their Cities

Journal name: International Journal of Health Policy and Management (IJHPM)

Authors' information: Celia Burgaz[1,2]*, Iris Van Dam[1], Adama Diouf[3], Kouakou Kouakou Philipps[4], Olouwafemi M. Mama[3], Sabiba Kou'santa Amouzou[5], Rebecca Rachel Assa Yao[4], Blessing Atwine[6], Madina M. Guloba[6], Lallepak Lamboni[5], Pauline Nakitende[6], Julien S. Manga[7], Clémence Metonnou[8], Célestin Koffi N'dri[4], Reynald Santos[8], Charles Sossa[8], Papa M.D.D. Sylla[9], Tiatou Souho[5], Stefanie Vandevijvere[1]

[1]Department of Epidemiology and Public Health, Sciensano, Brussels, Belgium.

[2]Department of Geosciences, Environment and Society, Université libre de Bruxelles (ULB), Brussels, Belgium.

[3]Laboratoire de Recherche en Nutrition et Alimentation Humaine (LARNAH), Université Cheikh Anta Diop, Dakar, Senegal.

[4]Université Alassane Ouattara (UAO), Bouaké, Côte d'Ivoire.

[5]Laboratoire de Biochimie des Aliments et Nutrition, University of Kara, Kara, Togo.

[6]Economic Policy Research Centre (EPRC), Kampala, Uganda.

[7]Department of Nutrition, University of Montreal, Montreal, QC, Canada.

[8]Regional Institute of Public Health, Université of Abomey-Calavi (UAC), Ouidah, Benin.

[9]Laboratoire des Sciences Biologiques, Agronomiques, Alimentaires et de Modélisation des Systèmes Complexes (LABAAM), Université Gaston Berger de Saint-Louis, Saint-Louis, Senegal.

***Correspondence to:** Celia Burgaz; Email: celia.burgaz@sciensano.be

Citation: Burgaz C, Van Dam I, Diouf A, et al. Barriers and facilitators to the development and implementation of public policies addressing food systems in five sub-Saharan African countries and five of their cities. Int J Health Policy Manag. 2025;14:8592. doi:10.34172/ijhpm.8592

Supplementary file 1. Definition of Key Terms and Ethic Approvals

Outcomes

Undernutrition: Undernutrition encompasses food insecurity (lack of regular access to enough food), stunting (low height-for-age), wasting (low weight-for-height), underweight (low weight-for-age), and micronutrient deficiencies (e.g. iron, vitamin A, and iodine) (B. A. Swinburn et al., 2019). In this article, the term is used to refer to child and maternal undernutrition as part of malnutrition in all its forms.

Obesity: Obesity is defined as a BMI >30 kg/m2, but when we refer to obesity. In this article, the term is used to encompass high body mass index (BMI) and non-communicable disease (NCD) dietary risks that form part of malnutrition in all its forms (B. A. Swinburn et al., 2019).

Climate change: Technically speaking, the term refers to the change in the state of the climate that persists for an extended period, typically, for decades or longer (IPCC, 2018). However, the term 'climate change' is used in this article to keep the wording used to describe the Global Syndemic (B. A. Swinburn et al., 2019). However, it also includes other factors related to environmental degradation, that go beyond climate change. These aspects are related to the process through which the natural environment is compromised in some way (e.g. water use, land use, eutrophication), reducing biological diversity (i.e. biodiversity, vegetation) and the general health of the environment (GEMET, 2024).

Domains and subdomains

Food supply chains: The complex journey of food, that comprises food production, storage, and delivery of food to reach the final consumer by the due date (Zhong et al., 2017). In this thesis, the term is used to refer to the stages that food/drinks go through, from the farm or sea, to the final consumer. It includes aspects such as agriculture, storage, processing, packaging, distribution, and trade agreements among countries. It also includes the processes of food loss and food waste.

Food environments: The collective physical, economic, policy and socio-cultural surroundings, opportunities and conditions that influence people's food and beverage choices and nutritional status (B. Swinburn et al., 2013). In this thesis, the term is used to refer to all the factors that influence the consumer to choose specific food/drinks. It includes aspects such as composition, labelling, promotion, provision, retail and prices.

Food production: The term is used in this thesis to describe the initial production stage activities to source crops, livestock and fish (i.e. agriculture, aquaculture and fisheries).

Food storage, processing, packaging and distribution: The term is used in this thesis to describe the preparation, storage and processing that the crops, livestock and fish undergo once the product has been harvested. Once the food/drink has been transformed and packaged, it is transported and distributed to the suppliers or retailers.

Food loss and waste: The term is used in this thesis to describe the amount of food/drink products that are produced but are not consumed. Food loss refers to the decrease in edible food mass at the

production, post-harvest and processing stages. Food waste refers to the food/drinks available for consumption that are disposed of either at the retail or consumer levels.

Food trade: The term is used in this thesis to describe the international treaties or trade conditions between countries, for food/drink products and their associated services.

Food composition: The term is used in this thesis to describe the reformulation of food/drink products by the industry, to change the mix of the product ingredients (salt, saturated fat, trans fat, added sugar) to try to make them healthier and/or more environmentally sustainable.

Food labelling: The term is used in this thesis to describe the information available to consumers on the packaging of food/drink products or menu boards in restaurants to understand their nutritional value and/or environmental impact.

Food promotion: The term is used in this thesis to describe the marketing strategies for food/drink products that increase their appeal among consumers. It includes advertisements across all media (e.g. television, radio, internet), schools, public transport, sports halls, etc.

Food provision: The term is used in this thesis to describe the provision of food/drink products in government-funded settings (e.g., hospitals, schools, universities, elderly homes, prisons, military canteen).

Food retail: The term is used in this thesis to describe the availability of food/drink products within communities (outlet density and location) and in-store (product placement).

Food prices: The term is used in this thesis to describe the affordability and/or accessibility of food/drinks to consumers (e.g., taxes, subsidies, food banks).

References

GEMET. (2024). *Environmental degradation—Definition*. https://www.eionet.europa.eu/gemet/en/concept/15154

IPCC. (2018). *Annex I: Glossary [Matthews, J.B.R. (ed.)]. In: Global Warming of 1.5°C. An IPCC Special Report on the impacts of global warming of 1.5°C above pre-industrial levels and related global greenhouse gas emission pathways, in the context of strengthening the global response to the threat of climate change, sustainable development, and efforts to eradicate poverty*. Annex I: Glossary [Matthews, J.B.R. (Ed.)]. In: Global Warming of 1.5°C. An IPCC Special Report on the Impacts of Global Warming of 1.5°C above Pre-Industrial Levels and Related Global Greenhouse Gas Emission Pathways, in the Context of Strengthening the Global Response to the Threat of Climate Change, Sustainable Development, and Efforts to Eradicate Poverty. [Masson-Delmotte, V., P. Zhai, H.-O. Pörtner, D. Roberts, J. Skea, P.R. Shukla, A. Pirani, W. Moufouma-Okia, C. Péan, R. Pidcock, S. Connors, J.B.R. Matthews, Y. Chen, X. Zhou, M.I. Gomis, E. Lonnoy, T. Maycock, M. Tignor, and T. Waterfield (Eds.)]. Cambridge University Press, Cambridge, UK and New York, NY, USA, Pp. 541-562. https://www.ipcc.ch/sr15/chapter/glossary/

Swinburn, B. A., Kraak, V. I., Allender, S., Atkins, V. J., Baker, P. I., Bogard, J. R., Brinsden, H., Calvillo, A., De Schutter, O., Devarajan, R., Ezzati, M., Friel, S., Goenka, S., Hammond, R. A., Hastings, G., Hawkes, C., Herrero, M., Hovmand, P. S., Howden, M., … Dietz, W. H. (2019). The Global Syndemic of Obesity, Undernutrition, and Climate Change: The Lancet Commission report. *The Lancet*, *393*(10173), 791–846. https://doi.org/10.1016/S0140-6736(18)32822-8

Swinburn, B., Sacks, G., Vandevijvere, S., Kumanyika, S., Lobstein, T., Neal, B., Barquera, S., Friel, S., Hawkes, C., Kelly, B., L'abbé, M., Lee, A., Ma, J., Macmullan, J., Mohan, S., Monteiro, C., Rayner, M., Sanders, D., Snowdon, W., … INFORMAS. (2013). INFORMAS (International Network for Food and Obesity/non-communicable diseases Research, Monitoring and Action Support): Overview and key principles. *Obesity Reviews: An Official Journal of the International Association for the Study of Obesity*, *14 Suppl 1*, 1–12. https://doi.org/10.1111/obr.12087

Zhong, R., Xu, X., & Wang, L. (2017). Food supply chain management: Systems, implementations, and future research. *Industrial Management & Data Systems*, *117*(9), 2085–2114. https://doi.org/10.1108/IMDS-09-2016-0391

Details of the ethics approvals for this study, with committees and reference numbers and team members per country.

Country	Ethical committee and country teams	Reference number
**Benin**	Local Ethics Committee for Biomedical Research of the University of Parakou, Benin<br>Country team:<br>• Clémence G. Metonnou Adanhoume<br>• Reynald Santos<br>• Charles Sossa	0574/CLERB-UP/P/SP/R/SA
**Côte d'Ivoire**	Comité National d'Ethique des Sciences de la Vie et de la Santé (CNESVS)<br>Country team:<br>• Kouakou Philipps Kouakou<br>• Rebecca Rachel Assa Epse Yao<br>• Célestin Koffi N'dri	IRB00011917
**Senegal**	Comité National d'Ethique pour la Recherche en Santé (CNERS), Ministère de la Santé et de l'Action Sociale (MSAS)<br>Country team:<br>• Olouawafemi M. Mama<br>• Adama Diouf<br>• Julien Soliba	00000103 MSAS/CNERS/SP

	• Papa Mamadou Dit Doudou Sylla	
**Togo**	Comité de Bioéthique pour la Recherche en Santé (CBRS)<br>Country team:<br>• Emile Sabiba Kousanta Amouzou<br>• Tiatou Souho<br>• Lallepak Lamboni	027/2023/CBRS
**Uganda**	College of Health Sciences, School of Public Health-Higher Degrees, Research and Ethics Committee (HDREC)<br>Country team:<br>• Madina M. Guloba<br>• Pauline Nakitende<br>• Blessing Atwine	IRB00011353<br>study protocol 860
