# Supplementary material for: Barriers and Facilitators to the Development and Implementation of Public Policies Addressing Food Systems in Five Sub-Saharan African Countries and Five of Their Cities
Source: Int J Health Policy Manag. 2025 Mar 18;14:8592. doi: 10.34172/ijhpm.8592 (PMC12089831; doi:10.34172/ijhpm.8592)
Supplement: Supplementary file 2 — Interview Guide. [file ijhpm-14-8592-s002.pdf]

Article title: Barriers and Facilitators to the Development and Implementation of Public Policies Addressing Food Systems in Five Sub-Saharan African Countries and Five of Their Cities

Journal name: International Journal of Health Policy and Management (IJHPM)

Authors' information: Celia Burgaz[1,2]*, Iris Van Dam[1], Adama Diouf[3], Kouakou Kouakou Philipps[4], Olouwafemi M. Mama[3], Sabiba Kou'santa Amouzou[5], Rebecca Rachel Assa Yao[4], Blessing Atwine[6], Madina M. Guloba[6], Lallepak Lamboni[5], Pauline Nakitende[6], Julien S. Manga[7], Clémence Metonnou[8], Célestin Koffi N'dri[4], Reynald Santos[8], Charles Sossa[8], Papa M.D.D. Sylla[9], Tiatou Souho[5], Stefanie Vandevijvere[1]

[1]Department of Epidemiology and Public Health, Sciensano, Brussels, Belgium.

[2]Department of Geosciences, Environment and Society, Université libre de Bruxelles (ULB), Brussels, Belgium.

[3]Laboratoire de Recherche en Nutrition et Alimentation Humaine (LARNAH), Université Cheikh Anta Diop, Dakar, Senegal.

[4]Université Alassane Ouattara (UAO), Bouaké, Côte d'Ivoire.

[5]Laboratoire de Biochimie des Aliments et Nutrition, University of Kara, Kara, Togo.

[6]Economic Policy Research Centre (EPRC), Kampala, Uganda.

[7]Department of Nutrition, University of Montreal, Montreal, QC, Canada.

[8]Regional Institute of Public Health, Université of Abomey-Calavi (UAC), Ouidah, Benin.

[9]Laboratoire des Sciences Biologiques, Agronomiques, Alimentaires et de Modélisation des Systèmes Complexes (LABAAM), Université Gaston Berger de Saint-Louis, Saint-Louis, Senegal.

***Correspondence to:** Celia Burgaz; Email: celia.burgaz@sciensano.be

Citation: Burgaz C, Van Dam I, Diouf A, et al. Barriers and facilitators to the development and implementation of public policies addressing food systems in five sub-Saharan African countries and five of their cities. Int J Health Policy Manag. 2025;14:8592. doi:10.34172/ijhpm.8592

Supplementary file 2. Interview Guide

Interview guide

Semi-structured interviews with city-level and national stakeholders

To assess the perceived barriers and facilitators for food systems policy development and implementation [1–9]

Interview topics and themes

**TOPIC**	**Food supply chains**	**Food environments**
THEME	Food production – area: Natural resources (e.g. soil, water, air, biodiversity)	Food composition
	Food production – area: Input resources (e.g. subsidies, fertilizers, pesticides, training/technology)	Food labelling
	Food production – area: Social aspects (e.g. support younger farmers, women empowerment)	Food promotion
	Food storage, processing, packaging and distribution	Food provision
	Food trade and investment	Food retail
	Food loss	Food prices
		Food waste

During the opening questions, after the introduction, it should become clear which topic(s) and theme(s) the stakeholder is working on. This info will then be used during the main questions.

Introduction

- Shortly introduce yourself (e.g. where do you work, how you are involved in the project,…).
- Thank stakeholder for participation.
- Explain the research project (e.g. how the indicators were developed and how the national and local policy mapping was conducted, as well as how the final policy index is going to be used in the future).
- Explain the aim of the interview.
  - *Interested in the barriers and facilitators for food system policy development at the appropriate level of jurisdiction (local/national).*
  - *Interested in both binding policies - laws, regulations, national strategic plans – and non-binding policies – guidelines, action plans, program implementation strategies.*
  - *Interview should take around 60 minutes.*
- Explain informed consent and ask stakeholder to sign.

   - *Make sure that the stakeholder has your contact details if any additional questions would come up later.*
   - *Explain that the audio recorder can be paused at any time during the interview.*
   - *Explain that the stakeholder can request for any parts of the audio recordings to be removed after the interview, up to [date].*
   - *Explain that the results will only be described and reported at an aggregated level.*
- Before proceeding, ask if there are any questions about the research project, the interview or the confidentiality.

Ask permission to turn on the recorder.

Opening questions

(+probing questions – to be asked depending on the first answer of the stakeholder)

I would like to start by getting a better insight in the work you do related to food systems policy development and/or implementation.

- Could you tell me a bit more about your tasks and responsibilities throughout the policy development and/or implementation process?
   - *Are you more involved in policy development or rather policy implementation? Could you explain a little further.*
   - *What is your role at the organisation you work? Could you elaborate on this.*
   - *What projects, organisations, ministries or institutes are you mainly reporting to? Could you go into a little more detail?*
- What policy areas are your main focus?
   - *If mentions themes within 'Food supply chains'* - *ask about other themes within this topic.*
   - *If mentions themes within 'Food environments'* - *ask about other themes within this topic.*

Obtain the topic and theme(s) the stakeholder is working on. This is essential to know before moving on to the main questions.

Main questions

(+probing questions – to be asked depending on the first answer of the stakeholder)

Main questions related to existing policies

– To be skipped if no policies related to the stakeholder's topic and theme(s) were identified during the national and local policy mapping.

Now I would like to know a bit more about the existing policies related to [topic] at the local/national level of jurisdiction.

Focus on the topic and theme(s) the stakeholder is working on and in which policies were identified during the national and local policy mapping.

- As far as you are aware, which policies are currently in place regarding [topic]?
  - *If stakeholder active on topic 'Food supply chains' – ask about different themes (based on the national and local policy mapping).*
  - *If stakeholder active on topic 'Food environments' - ask about different themes (based on the national and local policy mapping).*
- What might have facilitated policy development and implementation in the area of [topic and/or theme mentioned in previous question]?
  - *What might have been the key facilitator(s) that ensured [theme] policy development and implementation? Could you tell me a bit more?*
  - *Did any ministries/institutions/other stakeholders play an important role? How so?*
  - *Were there any specific local or national issues that led to the development and implementation of these policies? Or were there any global concerns?*
- Are you aware of any issues/barriers that hampered or slowed down either policy development or implementation in the area of [topic and/or theme mentioned in first questions]?
  - *Where there any actors or groups that actively opposed the policy development and/or implementation? How so? – e.g. Public opinion; Economic conditions; Scientific findings; Technological change; Interest groups such as NGOs or industry.*
  - *Where there any legal, institutional or jurisdictional barriers? Please elaborate.*
- Are there any lessons learnt (from the development and/or implementation of this policy) that might facilitate the development and implementation of similar policies in other countries or contexts? *Please explain a little further.*
- To what extent do you think that current [topic and/or theme mentioned in first questions] policies are effective?
  - *How could the current policies be improved?*
  - *What elements of these policies could be improved for them to be potentially more effective towards improving population nutrition/environmental sustainability?*

<u>Main questions related to non-existing policies</u>

During this (next) set of questions I would like to focus on <u>the policy development process</u> in regards to [topic].

Focus on the topic and theme(s) the stakeholder is working on, as obtained in the beginning of the interview. Ask these questions for all the different themes the stakeholder is working on.

- Which ministries/institutions/other stakeholders are involved in the development of [theme] policies?
  - *What is the role of the different ministries/institutions/other stakeholders involved?*

- *How do the different ministries/institutions/other stakeholders collaborate throughout the policy development process?*
- *Which other stakeholders do you think could or should be involved in the formulation of the [theme] policies? Why is this?*
- *What is the level of jurisdiction of the different ministries/institutions/other stakeholders involved?*

- What factors might facilitate [theme] policy development?
  - *Are there any broader political factors that might facilitate policy development? – e.g. Public opinion; Economic conditions; Scientific findings; Technological change; Interest groups such as NGOs or industry. Please explain in a little more detail.*
  - *What type of local or national issues could facilitate the development of [theme] policies? How so?*
- What factors might hamper [theme] policy development?
  - *Are there any broader political factors that might hamper policy development? – e.g. Public opinion; Economic conditions; Scientific findings; Technological change; Interest groups such as NGOs or industry. Please explain in a little more detail.*
  - *What type of local or national issues could hamper the development of [theme] policies? How so?*
- Towards the future, which are the most important actions you think the government can take in the area of [theme]?
  - *What factors might facilitate such actions? Why is this?*
  - *Do you have an idea of why such actions have not yet been taken? Please explain.*

Ending questions

(+probing questions – to be asked depending on the first answer of the stakeholder)

Before moving to the end of this interview I would like to ask you...

- Is there anything else important related to [theme] policies in [city/country] that we have not touched upon?
- In your experience, are there any additional policies that we have not discussed during this interview that should be developed to create sustainable food systems?

Thank you very much for your time. This is the end of our discussion for today.

Turn off the recording.

Before leaving, ask the stakeholder if there is anything else he/she would like to know or ask.

References

1. Plested, B. A., Edwards, R. W. & Jumper-Thurman, P. A Handbook for Successful Change. *Fort Collins: Tri-Ethnic Center for Prevention Research* (2006).
2. Rose, N., Reeve, B. & Charlton, K. Barriers and Enablers for Healthy Food Systems and Environments: The Role of Local Governments. *Curr Nutr Rep* (2022) doi:10.1007/s13668-022-00393-5.
3. Phulkerd, S., Sacks, G., Vandevijvere, S., Worsley, A. & Lawrence, M. Barriers and potential facilitators to the implementation of government policies on front-of-pack food labeling and restriction of unhealthy food advertising in Thailand. *Food Policy* **71**, 101–110 (2017).
4. Health Policy Project. *Capacity Development Resource Guide: Implementation Barriers*. 6 https://www.healthpolicyproject.com/pubs/272_ImplementationBarriersResourceGuide.pdf (2014).
5. Mohamed, S. F., Juma, P., Asiki, G. & Kyobutungi, C. Facilitators and barriers in the formulation and implementation of tobacco control policies in Kenya: a qualitative study. *BMC Public Health* **18**, 1–14 (2018).
6. Clarke, B. Understanding obesity prevention policy decision-making processes: a case study of Healthy Together Victoria using political and system sciences. (2018).
7. Gittell, R., Magnusson, M. & Merenda, M. *The sustainable business case book - Chapter 3.1 Factors That Influence Public Policy*. (The Saylor Foundation, 2012).
8. Meiksin, R. *et al.* Restricting the advertising of high fat, salt and sugar foods on the Transport for London estate: Process and implementation study. *Social Science & Medicine* **292**, 114548 (2022).
9. Kallio, H., Pietilä, A., Johnson, M. & Kangasniemi, M. Systematic methodological review: developing a framework for a qualitative semi-structured interview guide. *Journal of advanced nursing* **72**, 2954–2965 (2016).
