# Supplementary material for: Barriers and Facilitators to the Development and Implementation of Public Policies Addressing Food Systems in Five Sub-Saharan African Countries and Five of Their Cities
Source: Int J Health Policy Manag. 2025 Mar 18;14:8592. doi: 10.34172/ijhpm.8592 (PMC12089831; doi:10.34172/ijhpm.8592)
Supplement: Supplementary file 3 — List of Documents Provided by Each Country. [file ijhpm-14-8592-s003.pdf]

Article title: Barriers and Facilitators to the Development and Implementation of Public Policies Addressing Food Systems in Five Sub-Saharan African Countries and Five of Their Cities

Journal name: International Journal of Health Policy and Management (IJHPM)

Authors' information: Celia Burgaz[1,2]*, Iris Van Dam[1], Adama Diouf[3], Kouakou Kouakou Philipps[4], Olouwafemi M. Mama[3], Sabiba Kou'santa Amouzou[5], Rebecca Rachel Assa Yao[4], Blessing Atwine[6], Madina M. Guloba[6], Lallepak Lamboni[5], Pauline Nakitende[6], Julien S. Manga[7], Clémence Metonnou[8], Célestin Koffi N'dri[4], Reynald Santos[8], Charles Sossa[8], Papa M.D.D. Sylla[9], Tiatou Souho[5], Stefanie Vandevijvere[1]

[1]Department of Epidemiology and Public Health, Sciensano, Brussels, Belgium.

[2]Department of Geosciences, Environment and Society, Université libre de Bruxelles (ULB), Brussels, Belgium.

[3]Laboratoire de Recherche en Nutrition et Alimentation Humaine (LARNAH), Université Cheikh Anta Diop, Dakar, Senegal.

[4]Université Alassane Ouattara (UAO), Bouaké, Côte d'Ivoire.

[5]Laboratoire de Biochimie des Aliments et Nutrition, University of Kara, Kara, Togo.

[6]Economic Policy Research Centre (EPRC), Kampala, Uganda.

[7]Department of Nutrition, University of Montreal, Montreal, QC, Canada.

[8]Regional Institute of Public Health, Université of Abomey-Calavi (UAC), Ouidah, Benin.

[9]Laboratoire des Sciences Biologiques, Agronomiques, Alimentaires et de Modélisation des Systèmes Complexes (LABAAM), Université Gaston Berger de Saint-Louis, Saint-Louis, Senegal.

***Correspondence to:** Celia Burgaz; Email: celia.burgaz@sciensano.be

Citation: Burgaz C, Van Dam I, Diouf A, et al. Barriers and facilitators to the development and implementation of public policies addressing food systems in five sub-Saharan African countries and five of their cities. Int J Health Policy Manag. 2025;14:8592. doi:10.34172/ijhpm.8592

Supplementary file 3. List of Documents Provided by Each Country

Benin

1. Arrêté interministériel N°422/MAEP/MS/MICPME/MEF/MCAAT/MDGLAAT/DC/SGM/DANA de 2013 fixant les conditions de production, d'importation, de commercialisation et d'utilisation du sel iodé en République du Bénin.
2. Arrêté interministériel N°0237MS/MEF/MAEP/MICPME/DC/SGM/CTJ/DSME/SA de 2012 qui précise les modalités de fortification en fer, en zinc, en vitamines B et en acide folique de toute farine de blé destinée à la consommation humaine et animale et République du Bénin
3. Arrêté interministériel N°238 MS/MEF/MAEP/MICPME/DC/SGM/CTJ/DSME/SA de 2012 qui précise les modalités de fortification en Vitamine A, des huiles alimentaires destinées à la consommation humaine et animale en République du Bénin
4. Arrêté interministériel portant organisation, attribution et fonctionnement des comités et conseils de pêche en république du Bénin
5. Arrété N° 084 portant annulation de l'enregistrement de l'association des pratiquants de la technique de pêche TOKPOKONOU
6. Code Général des impôts 2023
7. DECRET N 2OO7-283 DU 16 JUIN 2OO7 Portant création, attributions, organisation et fonctionnement du Conseil National de Transport Rural
8. DECRET N° 2014-100 DU 31 JANVIER 2014 portant création, attributions, organisation et fonctionnement du Fonds National de Développement Agricole (FNDA)
9. Décret n° 2015-029 du 29 janvier 2015 fixant les modalités d'acquisition des terres rurales en République du Bénin
10. Décret N° 2022-390 du 13 Juillet 2022 portant organisation des procédures de l'évaluation environnementale et sociale en République du Bénin
11. Décret N°85-242 du 14 Juin 1985 relatif à l'étiquetage et la présentation des denrées alimentaires
12. Décret nº 97-643 portant réglementation de la commercialisation des substituts du lait maternel et des aliments pour nourrissons.
13. Dispositions légales et règlementaires en matière d'utilisation des engrais chimiques visent une protection de l'environnement et par conséquent de la santé des populations
14. Guide méthodologique de restauration des ecosystemes de zones humides du Bénin
15. Loi – Cadre N°2014-19 du 07 Août 2014 relative à la Pêche et à l'aquaculture en République du Bénin
16. Loi 2016-25 portant organisation de la concurrence en République du Bénin
17. Loi Cadre sur l'environnement
18. Loi n° 2007·21 du 16 octobre 2007 Portant protection du consommateur en République du Bénin.
19. Loi N° 2015-07 du 20 Mars 2015 portant code de l'information et de la communication en République du Bénin
20. Loi n° 84-009 du 15 mars 1984 sur le contrôle des denrées alimentaires.
21. Mission de supervision du Projet d'Appui à la Diversification Agricole - PADA (Don IDA-H6550 & Crédit IDA-48840) et de préparation de son financement additionnel

22. Neutralité de la dégradation des terres, un moyen de mise en œuvre des conventions de Rio et des ODD au Bénin
23. Ordonnance portant réclamation générale de la pêche dans les eaux continentales du Dahomey
24. Plan de developpement agricole du pôle 7 2018 – 2021 Agence Territoriale de Développement Agricole (ATDA) OUEME-ATLANTIQUE-LITTORAL-MONO
25. Plan national d'adaptation aux changements climatiques du Bénin Ministère du Cadre de Vie et du Développement Durable Direction Générale de l'Environnement et du Climat (DGEC)
26. Plan National d'Investissements Agricoles et de Sécurité Alimentaire et Nutritionnelle (PNIASAN 2017 - 2021)
27. Plan Stratégique de Développement du Secteur Agricole (PSDSA)
28. Plan Stratégique de Relance du Secteur Agricole (PSRSA)
29. Plan stratégique opérationnel des actions d'alimentation et de nutrition dans le secteur agricole
30. Politique du Secteur Santé pour la Nutrition 2016 -2025
31. Politique Nationale de d'Alimentation Scolaire
32. Politique Nationale de la Jeunesse
33. Politique Nationale de Promotion du Genre au Bénin
34. Programme d'Action du Gouvernement 2021 - 2026
35. Programme d'Appui au Développement Participatif de la Pêche Artisanale (PADPPA)
36. Programme National d'Alimentation Scolaire Intégré (PNASI)
37. Projet d'appui a la production vivriere et de renforcement de la resilience dans les departements de l'alibori, du borgou et des collines (papvire -abc)
38. Projet de document actualise de politique semenciere nationale du Bénin
39. Stratégie Nationale de l'Agriculture Sensible à la Nutrition (SNASN) et Plan d'Actions 2021-2025

Côte d’Ivoire

1. Accord entre le Programme Alimentaire Mondial (PAM), la Banque Alimentaire de Côte d'Ivoire et le Centre d'Excellence Régional contre la faim et la malnutrition.
2. Appui au Programme Intégré de pérennisation des Cantines Scolaires
3. Appui au renforcement des capacités et du cadre réglamentaire en matière de prévention/réduction des pertes après capture des produits halieutiques en Côte d'Ivoire.
4. Appui aux coopératives du secteur vivrier
5. Code forestier de la Côte d'Ivoire
6. Décret 96-894 déterminant les règles et procedures applicables aux études relatives à l'impact environnemental des projects de développement
7. Décret n°2013-416 du 6 juin 2013 portant réglementation de la commercialisation des substituts du lait maternel
8. Décret n°2016-1152 du 28 décembre 2016 rendant certaines normes d'application obligatoire
9. "Décret n°2017-567 portant approbation du programme vérification de la conformité des produits embarqués à destination de la Côte d'Ivoire.
10. Decret-n°92-487 du-26-aout-1992 portant Étiquetage et présentation des denrées alimentaires.
11. Dix ans après la Déclaration de Maputo sur l'agriculture et la Sécurité l'alimentaire : Une évaluation des progrès de la Côte d’Ivoire
12. Élaboration du plan de mise en œuvre des contributions déterminées au niveau national
13. Engagement de mettre en ouvre la Neutralité en matière de Dégradation des Terres
14. Étude de faisabilité de l'enrichissement du riz en Côte d'Ivoire
15. Fonds d'appui à l'entrepreneuriat des jeunes
16. Governance environnementale et développement durable en Côte d'Ivoire: initiaitve pour la durabilité des pêches
17. L'eau source de vie, préservons la
18. Loi n°2016-410 du 15 juin 2016 relative à la répression des fraudes et des falsifications en matière de vente des biens ou services
19. Loi n°91-1000 du 11 décembre 1991 portant interdiction et répression de la publicité mensongère ou trompeuse.
20. Plafonnement du prix des denrées alimentaires de grande consommation
21. Plan d’Investissement d’une agriculture intelligente face changement climatique
22. Plan de Gestion des Pestes (PGP)
23. Plan National de Développement (PND 2016-2020), Tome 1
24. Plan National de Développement (PND 2021-2025), Tome 2
25. Plan National Multisectoriel de Nutrition
26. Politique nationale de l'environnement et du développement durable
27. Politique Nationale de Promotion des petites et moyennes entreprises
28. Production Agricole et à la Commercialisation (PROPACOM)
29. Programme Filets Sociaux Productifs (PFSP)
30. Programme Intégré de pérennisation des Cantines Scolaires (PIP/CS)
31. Programme National Changement Climatique (PNCC)

32. Programme National d'Investissement Agricole de deuxième génération (2018-2025)
33. Programme social du Gouvernement (+ project d'autonomisation des femmes et dividende démographique au Sahel)
34. Progreamme d'appui au secteur vivrier en Côte d'Ivoire
35. Project de Restauration de Couvert Forestier (PRCF)
36. Project d'E-agriculture en Côte d'Ivoire
37. Projet de Développement durable des ressources génétiques du Tilapia du Nil dans le bassin de la Volta
38. PTBA 2022: Project d'accompagnement à la reconversion de 606 ex-militaires par la création de micro et petites entreprises dans le domaine agropastoral
39. Stratégie Nationale d'Alimentation Scolaire (SNAS)
40. Stratégie Nationale de conservation et d'utilisation durable de la diversité biologique de la Côte d'Ivoire
41. Stratégie nationale de gestion durable de la pêche - Politique nationale de Développement de l'Élevage, de la Pêche et de l'Aquaculture (PONADEPA)
42. Stratégie nationale de préservation, de réhabilitation et d'extension des forêts

Senegal

1. Charte du domaine irrigue de la vallee du fleuve senegal
2. Contribution Prévue Déterminée au niveau National 2016-2035
3. Décret 2010- 891 du 30 Juin 2010 portant création, organisation et fonctionnement du cadre national de commercialisation des productions agricoles
4. Décret N° 2004-1408 du 4 novembre 2004 portant création d'aires marines protégées
5. DECRET n° 2005-913 du 12 octobre 2005 portant application des normes Codex Alimentarius
6. Document de politique de santé/nutrition/ environnement dans le système éducatif
7. Document de politique foncière de 2016
8. Élaboration d'une proposition de loi visant à règlementer le marketing des substituts du lait maternel
9. Fonds d'autonomisation de la Délégation Générale à l'Entreprenariat Rapide des Femmes et des Jeunes (DER/FJ)
10. Guide des cantines scolaires : « investissons dans un environnement scolaire productif »
11. La loi no 2021-25 du 12 avril 2021 sur le prix et la protection du consommateur
12. Le Plan Stratégique de Fortification des Aliments en Micronutriments 2017-2021
13. Les Plans d'Occupation et d'Affectation des Sols (POAS) mis en œuvre dans la Vallée du Fleuve Sénégal
14. Lettre de Politique du Secteur de l'Environnement et du Développement Durable (LPSEDD) 2016-2020
15. Lettre de Politique Sectorielle de Développement 2016-2025
16. Lettre de Politique Sectorielle de Développement de l'Agriculture 2019-2023
17. Lettre de Politique Sectorielle de développement de la Pêche et de l'Aquaculture (LPSDPA) 2016 - 2023
18. Lettre de Politique Sectorielle de Développement de l'Agriculture (LPSDA) 2019 – 2023
19. Lettre de Politique Sectorielle de Développement du Ministère du Commerce, de la Consommation, du Secteur Informel et des PME (MCCSIPME) 2018-2022
20. Loi n° 2001 - 01 du 15 Janvier 2001 portant code de l'environnement
21. Loi Agro-sylvo- pastoral et halieutique 2004-2024
22. Loi n. 2022-06 du 15 avril 2022 portant Code de l'Aquaculture
23. Loi n° 2008/29 du 28 juillet 2008 portant loi d'orientation relative à la promotion et au développement des petites et moyennes entreprises.
24. Loi n° 2015-18 du 13 juillet 2015 portant Code de la Pêche maritime
25. Plan d'action de gestion intégrée des ressources en eau 2018-2030
26. Plan National d'Adaptation du Secteur de la peche et de l'aquaculture face au changement climatique horizon 2035
27. Plan Stratégique de Développement de la pêche continentale au Sénégal 2021-2025
28. Plan Stratégique Multisectoriel de la nutrition 2018-2022
29. PNDE-Plan National de Développement de l'Élevage 2016- 2025
30. Politique Forestière du Sénégal 2005-2025
31. Politique Nationale de Gestion des Zones Humides de 2015 (PNZH)
32. Programme Agricole pour la Souveraineté Alimentaire Durable 2021-2023

33. Programme d'Accélération de la Cadence de l'Agriculture Sénégalaise (PRACAS) 2014-2018
34. Programme d'Amélioration de la Qualité de l'Equité et de la Transparence-Education et Formation (PAQUET-EF) 2013-2025
35. Programme National d'appui à la Sécurité alimentaire et à la résilience 2018-2022
36. Programme National d'investissement Agricole pour la Sécurité Alimentaire et la Nutrition 2018 -2022
37. Programme National des Domaines Agricoles Communautaires 2014-2019
38. Programme pays 2018-2030
39. Programmen national de Bourse de Sécurité Familiale (PNBSF)
40. Projet d'Appui à l'Agriculture Irriguée et au Développement Economique de Podor (AIDEP)
41. Stratégie Nationale de Gestion des Aires Marines Protégées
42. Stratégie Nationale de Protection Sociale 2016-2035
43. Stratégie Nationale de Sécurité Alimentaire et de Résilience 2015-2035
44. Stratégie Nationale et Plan National d'Action pour la biodiversité 2015-2030

Togo

1. Arrete Interministeriel N° /Mapah/ Mcidsppcl/ Mctl Portant Valorisation Et Vulgarisation Des Mets Locaux
2. Arrêté interministériel n°133/2013MS/MCPSP/MIZFIT définissant les conditions d'applications du décret n°2012-010/PR relatif à l'enrichissement des huiles raffinées et de la farine de blé en micronutriments
3. Arrete N°012/Mdprcpsp/Dcic Portant Contrôle De Produits Et Service Objets De Publicite Commerciale Au Togo
4. Arrêté N°17/MAEP/SG/DEF portant interdiction d'importation de croupions de dinde
5. Arrêté N°183|19/MAPAH/CAB/SG/DPV portant interdiction de et d'utilisation du glyphosate et tout produit le contenant
6. Article 1 de la LOI N° 2008-005 PORTANT LOI-CADRE SUR L'ENVIRONNEMENT
7. Attributions de l'Institut de conseil et d'Appui Techniques (ICAT) - Politique sectorielle de la pêche et l'aquaculture
8. Cadre Strategique d'Investissements pour la Gestion de l'Environnement et des Ressources Naturelles au Togo (CSIGERN 2018-2022)
9. Décret N° 2012-031/PR portant création, attributions et fonctionnement du comité national des mesures sanitaires et phytosanitaires (SPS)
10. Decret N° 98-099/Pr Portant Application De La Loi N° 96-007 Du 03 Juillet 1996 Relative A La Protection Des Vegetaux
11. Instruction N°152/Mdn Sur L'organisation Et Le Fonctionnement Du Service De L'ordinaire Dans Les Corps De Troupes
12. Loi N°2008-005 Du 30 Mai 2008 Portant Loi-Cadre Sur L'environnement
13. Loi D'orientation Des Transport
14. Loi N° 99-011 Du Decembre 1999 Portant Organisation De La Concrurence Au Togo
15. Loi N°2008-005 Du 30 Mai 2008 Portant Loi-Cadre Sur L'environnement
16. Loi N°2009-007 Portant Code De La Sante Publique De La Republique Toglaise
17. Loi N°2016-026 Du 11/10/2016 Portant Reglementation De La Peche Et De L'aquaculture Au Togo
18. Loi N°2020-001 Du 07 Janvier 2020 Relative Au Code De La Presse Et De La Communication
19. Loi N°2020-007 Du 26 Juin 2020 Relative A L'alimentation Scolaire
20. Loi N°99-011 Du 28 Decembre 1999 Portant Organisation De La Concurrence Au Togo
21. Loi N°2010-004 Portant Code De I'eau
22. Mecanisme Forets Et Paysans de la FAO
23. Note de service Du Ministère de L'Agriculture pour la consommation locale des produits de volailles.
24. Plan D'actions National De Gestion Integree Des Ressources En Eau-Togo
25. Plan National D'adaptation Aux Changements Climatiques Au Togo (PNACC)
26. Plan Stratégique De Pays – Togo (2022–2026) du Programme Alimentaire Mondial
27. Politique Agricole Assortie Du Plan Strategique Pour La Transformation De L'agriculture Au Togo A L'horizon 2030 (PA-PSTAT 2030)

28. Politique Et Plan Strategique Multisectoriel De Lutte Contre Les Maladies Non Transmissibles 2018-2022
29. Politique national de l'environnement
30. Politique Nationale D'alimentation Scolaire Au Togo
31. Politique Nationale De L'alimentation Saine Et Equilibre En Milieux Scolaire, Extra-Scolaire Et Universitaire
32. Politique Nationale De L'alimentation Scolaire (PNAS)
33. Politique Nationale En Matiere D'alimentation Et De Nutrition
34. Politique Nationale Multisectorielle De Nutrition (PNMN, 2019-2030)
35. Politique sectorielle de la pêche et l'aquaculture
36. Programme d'Appui a la Lutte contre le Changement Climatique (PALCC)
37. Programme National D'investissement Agricole, De Securite Alimentaire Et Nutritionnelle – PNIASAN
38. Projet Appui A La Relance Du Secteur Semencier
39. Projet D 'appui Au Developpement Agricole Au Togo (PADAT)
40. Projet d'Appui à l'Employabilité et à l'Insertion des Jeunes dans les Secteurs Porteurs (PAEIJ-SP)
41. Projet D'appui Au Mecanisme Incitatif De Financement Agricole Fonde Sur Le Partage De Risques (Pro-MIFA)
42. Projet d'Appui aux Investissements Agricoles des Jeunes (PAIAJ)
43. Rapport d'activité de l'Institut de Conseil et d'Appui Technique (ICAT) de la Région Centrale Exercice 2022
44. Strategie De Developpement Du Secteur Semences Rizicoles (2016-2030)
45. Stratégie et Plan d'Action National pour la Biodiversité du Togo (SPANB) 2010-2020

Uganda

1. Agriculture Cluster Development Project
2. Agriculture Sector Strategic Plan 2015/16-2019/20
3. Bylaws on Riverbanks management.
4. Bylaws on Sustainable land management.
5. Emyooga Wealth Fund
6. Excise Duty Act
7. Mbale Counterfeit Agricultural Inputs (Prohibition) Bill
8. National Agricultural Extension Strategy (2016-2021)
9. National Agricultural Research Act
10. National Agriculture Policy
11. National Biodiversity Strategy and Action Plan
12. National Climate Change Policy
13. National Fertilizer Policy
14. National Grain Trade Policy
15. National Health Policy
16. National Irrigation Master Plan (2010-2035)
17. National Seed Policy
18. National Strategy for Youth Employment in Agriculture (2016-2021)
19. National Water Policy
20. Operation Wealth Creation (OWC)
21. Plan Protection and Health Act
22. The Advertising Standards Code (under the Uganda Communications Commission Act)
23. The Agricultural Chemicals (Control Act)
24. The Agriculture Cluster Development Project
25. The East African Community Customs Management Act
26. The East African Standard fortified edible oils and fats Specification
27. The Fertilizer Control Regulation
28. The Fish Aquaculture Rules
29. The Food and Drugs Act
30. The National Biotechnology and Biosafety Bill
31. The National Environment Management Policy
32. The National Environment Regulation (Miminum Standards for Management of Soil Quality)
33. The National Environment Standards for Discharge of Effluent into water or land Regulations
34. The National Fisheries and Aquaculture Policy
35. The National Land Use Policy
36. The National School Health Policy
37. The National Youth Manifesto 2016-2021
38. The National Youth Policy
39. The Plant Variety Protection Act
40. The Seeds and Plant Regulation

41. The Uganda Food and Nutrition Policy
42. The Uganda Food and Nutrition Strategy and Investment Plan
43. The Uganda Gender Policy
44. The Uganda Green Growth Development Strategy 2017/18 – 2030/31
45. The Uganda Multi-Sectoral Food Security and Nutrition Project (UMFSNP)
46. The Uganda National Bureau of Standards Act (Declaration of Compulsory National Standards) Notice
47. The Uganda National Land Policy
48. The Uganda Women Entrepreneurship Programme (UWEP)
49. The Ugandan National Land Policy
50. Uganda Micro, Small and Medium Enterprise (MSME) Policy
51. Uganda National Climate Change Policy
52. Uganda National Irrigation Policy
53. Uganda National Seed Strategy 2014/15-2019/20
54. Uganda Nutrition Action Plan 2011-2016
55. Uganda Rural Fund
56. Uganda Water Action Plan
57. USAID/Uganda Agricultural Sector Pesticide Procedures Guide (ASPPG)
58. Value Added Tax Act
59. Youth Livelihood Programme (YLP)
