# Supplementary material for: Barriers and Facilitators to the Development and Implementation of Public Policies Addressing Food Systems in Five Sub-Saharan African Countries and Five of Their Cities
Source: Int J Health Policy Manag. 2025 Mar 18;14:8592. doi: 10.34172/ijhpm.8592 (PMC12089831; doi:10.34172/ijhpm.8592)
Supplement: Supplementary file 4 — Thematic Codes Identified During the Analysis of the Interviews, and Reasons for Exclusion of Interviews. [file ijhpm-14-8592-s004.pdf]

Article title: Barriers and Facilitators to the Development and Implementation of Public Policies Addressing Food Systems in Five Sub-Saharan African Countries and Five of Their Cities

Journal name: International Journal of Health Policy and Management (IJHPM)

Authors' information: Celia Burgaz[1,2]*, Iris Van Dam[1], Adama Diouf[3], Kouakou Kouakou Philipps[4], Olouwafemi M. Mama[3], Sabiba Kou'santa Amouzou[5], Rebecca Rachel Assa Yao[4], Blessing Atwine[6], Madina M. Guloba[6], Lallepak Lamboni[5], Pauline Nakitende[6], Julien S. Manga[7], Clémence Metonnou[8], Célestin Koffi N'dri[4], Reynald Santos[8], Charles Sossa[8], Papa M.D.D. Sylla[9], Tiatou Souho[5], Stefanie Vandevijvere[1]

[1]Department of Epidemiology and Public Health, Sciensano, Brussels, Belgium.

[2]Department of Geosciences, Environment and Society, Université libre de Bruxelles (ULB), Brussels, Belgium.

[3]Laboratoire de Recherche en Nutrition et Alimentation Humaine (LARNAH), Université Cheikh Anta Diop, Dakar, Senegal.

[4]Université Alassane Ouattara (UAO), Bouaké, Côte d'Ivoire.

[5]Laboratoire de Biochimie des Aliments et Nutrition, University of Kara, Kara, Togo.

[6]Economic Policy Research Centre (EPRC), Kampala, Uganda.

[7]Department of Nutrition, University of Montreal, Montreal, QC, Canada.

[8]Regional Institute of Public Health, Université of Abomey-Calavi (UAC), Ouidah, Benin.

[9]Laboratoire des Sciences Biologiques, Agronomiques, Alimentaires et de Modélisation des Systèmes Complexes (LABAAM), Université Gaston Berger de Saint-Louis, Saint-Louis, Senegal.

***Correspondence to:** Celia Burgaz; Email: celia.burgaz@sciensano.be

Citation: Burgaz C, Van Dam I, Diouf A, et al. Barriers and facilitators to the development and implementation of public policies addressing food systems in five sub-Saharan African countries and five of their cities. Int J Health Policy Manag. 2025;14:8592. doi:10.34172/ijhpm.8592

Supplementary file 4. Thematic Codes Identified During the Analysis of the Interviews, and Reasons for Exclusion of Interviews

Key themes and subthemes identified and organised according to the categories of the Health Policy Triangle (HPT) framework.

**ACTORS**	Facilitator	Barrier
Individuals	14	5
Agrifood company or industry spokesperson	0	0
Celebrity	0	0
Civil servant	3	1
Independent opinion leader	2	1
Politician	12	5
Researcher or expert	3	1
Groups	31	17
Community group	16	2
Farmers or fishers	10	11
General public	19	11
Illegal groups	0	1
Indigenous groups	0	0
Police	1	1
Protest groups	0	0
Social movement	5	1
Youth	5	2
Organisations	36	26
Agrifood company or industry	12	10
International goverments	10	1
International organisation	20	1
Local government	11	4
Media	1	0
National government	33	20
NGOs	17	2
Private investors	1	1
Public sector agency	18	3
Religious organisation	4	0
Research institutes or academia	6	1
Trade platform or labour union	5	0

**CONTENT**	Facilitator	Barrier
Budget	17	20
Financial resources	17	19
Human resources and manpower	6	7
Clarity	15	15
Data	10	4
Definitions	2	4
Language	0	2
Responsibilities, coordination and accountability	3	6
Sustainability	3	5
Timelines	0	3
Communication	8	5
Accessibility of information	8	5
Official press releases	0	0
Knowledge capacity	5	8
Formation and competence	5	8
Policy objectives	21	6
Ecological objectives	8	2
Economic objectives	7	1
Health objectives	12	2
Social objectives	10	2
Policy type	14	6
Facilitating policies	9	1
Industry self-regulatory	0	0
Regulatory policies	6	3
Restrictive policies	2	2
Scope	9	7
Universally targeted	0	1
Selective targeted	2	0
Broad setting	5	5
Specific setting	3	3

**CONTEXT**	Facilitator	Barrier

**PROCESS**	Facilitator	Barrier

Cultural factors	3	6
Gender roles	1	1
Religion	0	0
Social behaviour	1	5
Traditions	1	1
International and exogenous factors	22	14
Climate change	8	1
Crisis, disease or pest outbreak	6	1
International agreements	12	4
Migration	0	1
Trade agreements	3	6
War and conflicts	2	2
Situational factors	30	21
Food insecurity	2	1
Government accountability	1	1
Political elections	0	3
Political stability	1	0
Political will	10	11
Population health	5	2
Public opinion and awareness	20	14
Scandals	1	0
Trust and transparency	3	3
Structural factors	17	24
Corruption and fraud	3	6
Economy	3	5
Infrastructure and mobility	4	4
Land use and planning	0	4
Laws	1	1
Political structure and bureaucracy	3	11
Poverty	0	3
Technology and innovation	5	3
Urbanisation	2	3

Agenda setting and problem definition	15	7
Problem awareness and agenda setting	15	7
Consultation	28	8
Interest groups	23	8
Scientific basis and research	11	0
Formulation and desing	8	5
Solutions or preferred options	8	5
Negotiation	21	16
Advocacy or lobbying	10	5
Cross-governmental	13	11
Partnerships	9	5
Adoption	1	5
Policy adoption	1	5
Implementation	3	17
Policy implementation	3	17
Validation and effectiveness	14	17
Policy monitoring and evaluation	14	17

Legend: The numbers refer to the number of interviews in which each code was identified, and the grading colour scale for all categories goes from 0 (the lowest value) to 20 or more (the highest value).

Reasons for exclusion of interviews

1. **Uganda_Local_1:** Agriculture expert. The whole interview did not address any barrier or facilitator nor for policy development or implementation. It explained the levels of jurisdiction in Mbale and some obstacles on the ground for farmers and food safety, but they were not related to policy. No policy information could be identified, as the stakeholder was not talking about anything specific to the policy index and none of the potential policies to implement.

2. **Cote d'Ivoire_National_5E:** In theory was a nutrition expert focusing on schools but it was changed for another colleague. This new stakeholder was working on agriculture but it was difficult to understand due to the language used and the context explained, which was not related to policy but to the individual school management. The interview was very long but they were not talking about policy, it was too specific for the local context and not at all related to policy development or implementation. It also felt as if the person conducting the interview was not following the questionnaire.

3. **Cote d'Ivoire_Local_14N:** In theory was going to be a female but they changed the expert. The expert worked in the regional office for employment and social affairs, but he was not working on anything related to the food system. He stressed many times that this was not something they worked on. He did not mention any kind of support for young people or gender equality, and only a few things mentioned were related to companies and dietitians at the local level giving recommendations on how to eat healthy. No mention of barriers or facilitators or any other type of information that can be useful for our research.
